# Isolable monoatomic monovalent bismuth complexes with a redox non-innocent bis-silylenyl carborane ligand†

**DOI:** 10.1039/d5sc02644j

**Published:** 2025-05-19

**Authors:** Jian Xu, Shenglai Yao, Verònica Postils, Eduard Matito, Christian Lorent, Matthias Driess

**Affiliations:** a Metalorganic and Inorganic Materials, Department of Chemistry, Technische Universität Berlin 10623 Berlin Germany matthias.driess@tu-berlin.de; b Theoretical Chemistry Group, Molecular Chemistry, Materials and Catalysis Division (MOST), Institute of Condensed Matter and Nanosciences, Université Catholique de Louvain Place Louis Pasteur 1 B-1348 Louvain-la-Neuve Belgium; c Donostia International Physics Center (DIPC) 20018 Donostia Euskadi Spain; d Ikerbasque Foundation for Science Plaza Euskadi 5 48009 Bilbao Euskadi Spain; e Physical and Biophysical Chemistry, Department of Chemistry, Technische Universität Berlin 10623 Berlin Germany

## Abstract

Utilizing the chelating bis(silylenyl)carborane [Si^II^(*closo*-CB)Si^II^] (A, Si^II^ = PhC(N*t*Bu)_2_Si, CB = *o*-C_2_B_10_H_10_) ligand, a series of unprecedented bis(silylene)-stabilized monovalent bismuth complexes {[Si^II^(*closo*-CB)Si^II^]Bi}X (X = I, 1a; X = OTf, 1b), {[Si^II^(*nido*-CB)Si^II^]Bi} (2) and ({[Si^II^(*nido*-CB)Si^II^]Bi}K(thf)_2_)_2_ ([3K(thf)_2_]_2_) were synthesized, isolated and characterized. The electronic structures of the bismuth complexes are significantly influenced by the redox-active nature of the CB scaffold. Remarkably, a one-electron injection to 1b with KC_8_ does not furnish a Bi^0^ complex but reduction of the CB backbone giving rise to the neutral Bi^I^ radical complex 2. Notably, compound 1b can also undergo a two-electron reduction with two molar equiv. of potassium naphthalenide, resulting in the formation of the diamagnetic Bi^I^ anion complex 3 as a dimer bridged *via* two K(thf)_2_ cations. Density functional theory calculations reveal that upon reduction from 1a to 2, and 2 to 3, the added electron predominantly localizes within the carborane cage, with a marked preference for the carbon atoms, ruling out that these species exhibit characteristics of a molecular bismuth(0) electride.

## Abstract 2

A series of unique bismuth(i) complexes supported by a bis(silylenyl)-*ortho*-carborane were synthesized and isolated. Their electronic structures are dominated by the redox-active character of the carborane scaffold.

## Graphical abstract

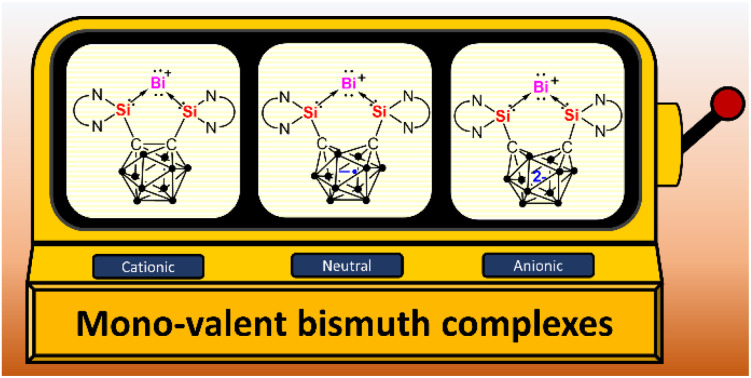

## Introduction

Bismuth, known since ancient times, is the heaviest stable element in the periodic table, possessing an extraordinarily long half-life of 1.9 × 10^19^ years.^1,2^ Despite its inherent stability, bismuth has been widely utilized in diverse research fields, including medical science, physical and materials sciences, and applied chemistry.^3–7^ In recent years, significant progress has been made in the study of low-valent bismuth compounds,^8–10^ highlighting their multiple accessible oxidation states and redox-active nature. These characteristics enable bismuth to participate in a variety of chemical transformations, presenting exciting opportunities for innovative catalytic applications.^11–13^ Furthermore, bismuth has emerged as a promising central ion in single-molecule magnets (SMMs) due to its strong spin–orbit coupling and stable multivalent states.^14–16^

**Fig. 1:**
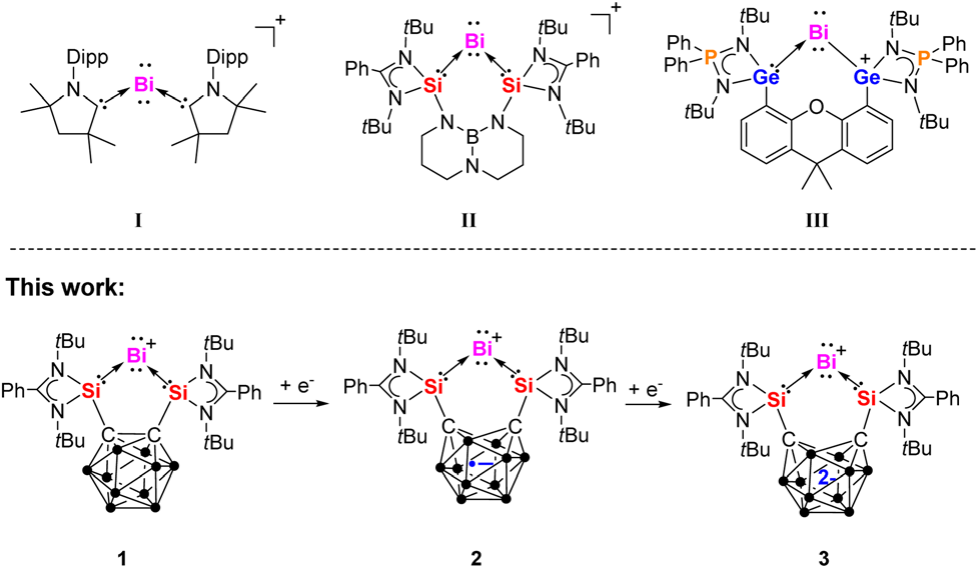
Known examples of cationic Bi^I^ complexes I–III and the Bi^I^ species 1–3 of this work bearing a redox non-innocent carborane cage.

Monoatomic zero-valent complexes of Group 14 elements, known as tetrylones, adopt the general formula L: → E^0^ ←: L (E = C, Si, Ge, Sn, Pb; L = Lewis donor ligands).^17–21^ Mono-valent cationic bismuth species can be regarded as the heaviest isoelectronic analogs of tetrylones, that is plumbylones, possessing two lone pairs on the central atom.^22,23^ The first cationic Bi^I^ compound (I, Fig. 1)^24^ was synthesized by reducing BiCl_3_ in the presence of cyclic alkyl(amino)carbenes (cAACs). Subsequently, the bis(silylene)-supported Bi^I^ cation complex [{Si^II^(TBD)Si^II^}Bi^I^][BAr^F^_4_] II (ref. 25) (Si^II^ = PhC(N*t*Bu)_2_Si, TBD = 1,8,10,9-triazaboradecalin, Ar^F^ = 3,5-(CF_3_)_2_-C_6_H_3_) was prepared through a one-pot reaction between the bis(silylene) {Si^II^(TBD)Si^II^} ligand and [IPr → BiBr_3_] (IPr = 1,3-bis(2,6-diisopropylphenyl)-imidazole-2-ylidene), followed by treatment with Na[BAr^F^_4_] in THF at −30 °C and reduction with two molar equiv. of potassium graphite (KC_8_). Recently, our group reported the bis(germylene)-supported cationic Bi^I^ complex III.^26^ Due to the redox non-innocent nature of the germylene moieties, the positive charge of the Bi^I^ cation migrates to one of the Ge atoms within the bis(germylene) ligand, resulting in the chelating germylium–germylene Bi^I^ complex III.

**Scheme 1:**
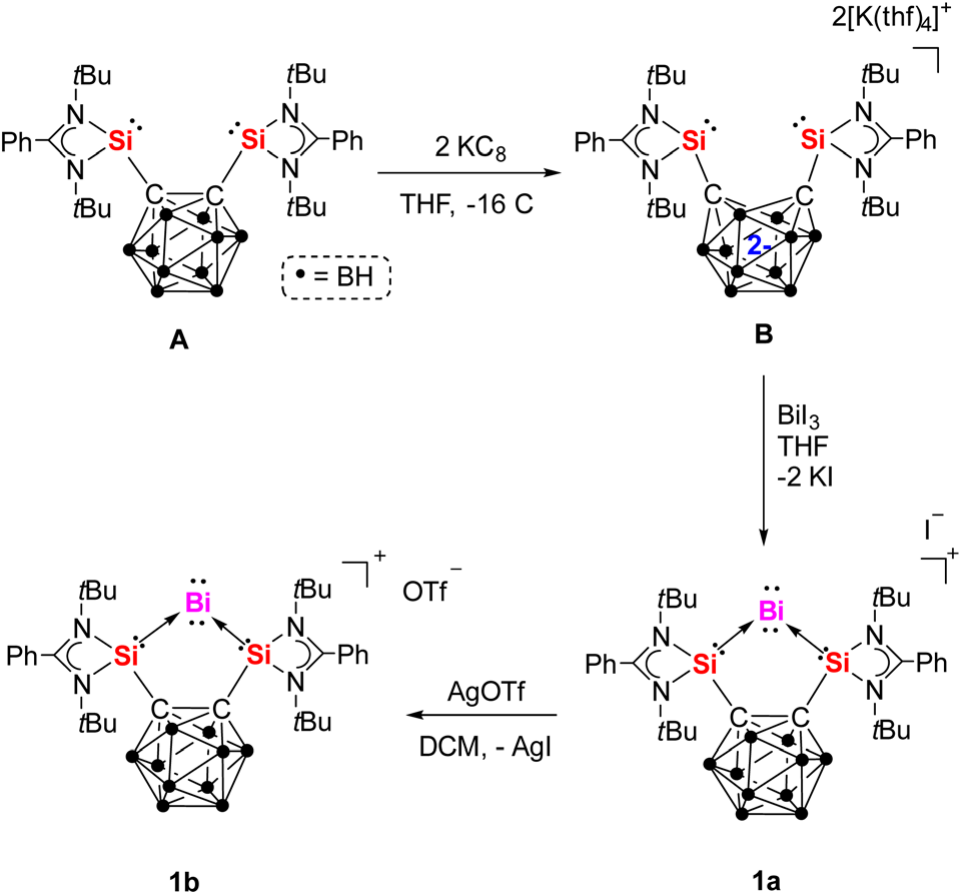
Synthesis of Bi^I^ cation complexes 1a and 1b from bis(silylenyl)-*o*-carborane A.

In recent years, our group has successfully designed and synthesized several chelating bis(NHSi) ligands (NHSi = *N*-heterocyclic silylene) featuring electronically and geometrically diverse spacers.^27^ These bis(NHSi) ligands have facilitated the isolation of various low-valent main-group compounds, including zero-valent group 14 and mono-valent group 15 complexes, which exhibit fascinating electronic structures and unique chemical reactivities.^28–35^ The bis(silylenyl)-*o*-carborane ligand [Si^II^(*closo*-CB)Si^II^] (A, Si^II^ = PhC(N*t*Bu)_2_Si, CB = *o*-C_2_B_10_H_10_)^36^ (Scheme 1), featuring a relatively short Si^II^⋯Si^II^ distance of approximately 3.3 Å, was first reported by our group in 2016. The latter ligand acts as a strong chelating Lewis donor due to the silylene moieties and exhibits interesting redox non-innocence attributed to the carborane spacer. These properties have proven effective in stabilizing monoatomic Si^0^ and Ge^0^ complexes^33,34^ as well as containing the isoelectronic N^I^ cation.^35^ Herein, we report the synthesis and characterization of the Bi^I^ cation complexes {[Si^II^(*closo*-CB)Si^II^]Bi}X (X = I, 1a; X = OTf, 1b) supported by the bis(silylenyl)-*o*-carborane A. Strikingly, the one-electron and two-electron reductions of 1b using KC_8_ and KC_10_H_8_, respectively, yield no Bi^0^ species but the neutral and anionic Bi^I^ complexes {[Si^II^(*nido*-CB)Si^II^]Bi} 2 and ({[Si^II^(*nido*-CB)Si^II^]Bi}K(thf)_2_)_2_ ([3K(thf)_2_]_2_), both featuring a *nido*-C_2_B_10_ core, yet in different reduced states. The electronic structures of this series of Bi^I^ complexes are further elucidated through Density Functional Theory (DFT) calculations.

## Results and discussion

### Synthesis of bis(silylene)-stabilized mono-valent single atom bismuth complexes

The dipotassium bis(silylenyl)-*nido*-dicarboranate precursor [{Si^II^(*nido*-CB)Si^II^}][K_2_(thf)_4_] (B),^33^ generated *in situ* from bis(silylenyl)-*o*-carborane (A) and two molar equivalents of KC_8_ in THF (Scheme 1), reacts with one equiv. of BiI_3_ at −30 °C to afford the Bi^I^ cation complex 1a. After workup, the desired Bi^I^ complex {[Si^II^(*closo*-CB)Si^II^]Bi}I (1a) was isolated as brown-yellow needle-shaped crystals in 57% yield. Subsequent treatment of 1a with one equiv. of AgOTf in dichloromethane (DCM) results in the replacement of the iodide counterion by OTf^−^, yielding the {[Si^II^(*closo*-CB)Si^II^]Bi}OTf complex 1b as an orange powder in 77% yield. The ^29^Si{^1^H} NMR spectra of 1a and 1b show singlets at *δ* = 68.7 and 66.9 ppm, respectively, both exhibiting significant downfield shifts relative to A (*δ* = 18.9 ppm).^28^

**Fig. 2:**
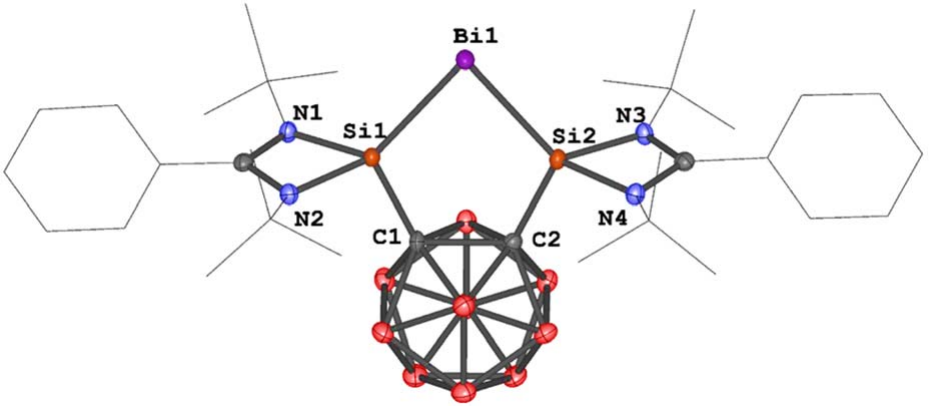
Molecular structures of the cations in 1a and 1b. Thermal ellipsoids are drawn at the 50% probability level. H atoms, anionic moieties and solvent molecules are omitted for clarity. Selected bond lengths (Å) and angles (deg.): 1a: Bi_1_–Si_2_ 2.5958(9), Bi_1_–Si_1_ 2.5940(10), C_2_–C_1_ 1.691(5), Si_2_–C_2_ 1.937(4), Si_1_–C_1_ 1.937(4), Si_1_–Bi_1_–Si_2_ 79.28(3), C_1_–Si_1_–Bi_1_ 115.14(11), C_2_–Si_2_–Bi_1_ 115.65(11). 1b: Bi_1_–Si_2_ 2.5774(6), Bi_1_–Si_1_ 2.5931(6), C_2_–C_1_ 1.692(3), Si_1_–C_1_ 1.923(2), Si_2_–C_2_ 1.925(2), Si_1_–Bi_1_–Si_2_ 79.486(18), C_1_–Si_1_–Bi_1_ 115.25(7), C_2_–Si_2_–Bi_1_ 115.53(7).

The molecular structures of 1a and 1b were determined by single-crystal X-ray diffraction (scXRD) analysis. Both exhibit a discrete ionic structure with a similar five-membered C_2_Si_2_Bi ring in the cation, where the central Bi^I^ site is coordinated to two silicon atoms. The Si–Bi bond lengths range from 2.5774(6) to 2.5958(9) Å (Fig. 2), similar to those in the [{Si^II^(TBD)Si^II^}Bi^I^][BAr^F^_4_] complex II (Fig. 1, 2.557(1) and 2.561(8) Å).^25^ The Si–C bond lengths span from 1.923(2) to 1.937(4) Å, while the C_1_–C_2_ distances in 1a and 1b [1.691(5) and 1.692(3) Å] are very close to that in A (1.71 Å).^36^ Notably, the Si_1_–Bi_1_–Si_2_ angles of 79.28(3)° and 79.486(18)° in 1a and 1b are slightly more acute than in complex II [82.10(3)°].^25^

**Scheme 2:**
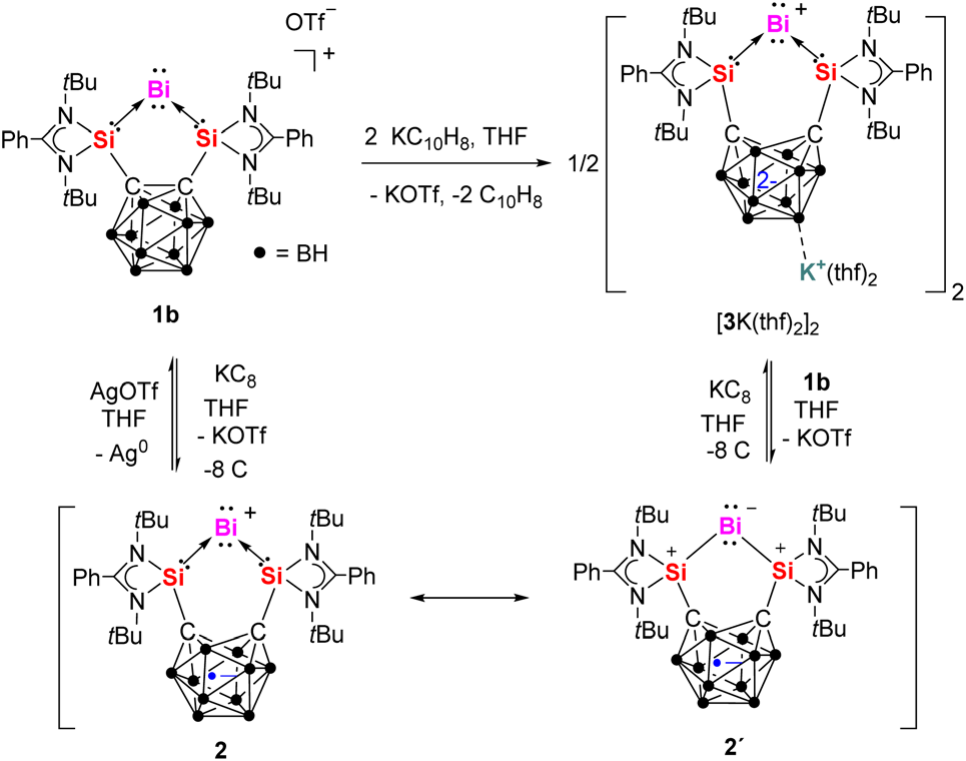
Reversible redox reactions between 1b, 2 (with its resonance structure 2′) and [3K(thf)_2_]_2_.

Notably, the cyclic voltammogram (CV) of 1b exhibits two quasi-reversible reduction processes at *E*_1/2_ = −1.36 V and −1.68 V *vs.* Fc/Fc^+^ (see ESI Fig. S11†). This prompted us to explore its chemical reduction. Upon mixing 1b with one equivalent of KC_8_ in THF at −30 °C, a deep-red solution formed, from which complex 2 was isolated in 43% yield (Scheme 2).

**Fig. 3:**
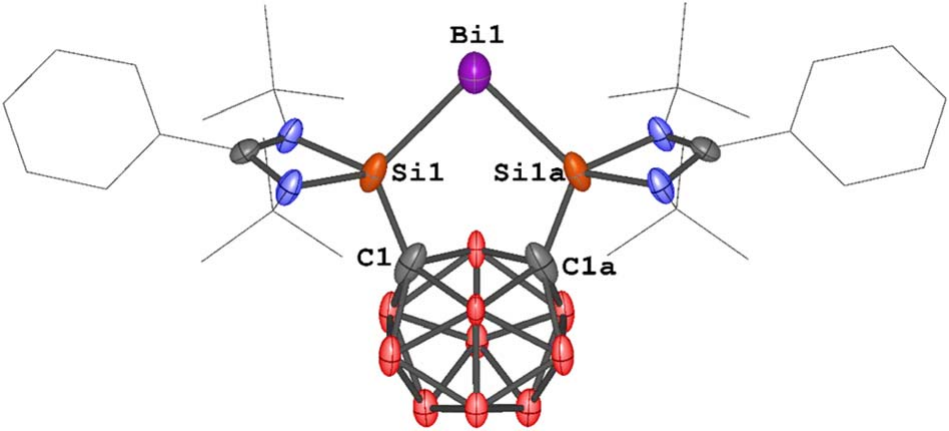
Molecular structure of 2. Thermal ellipsoids are drawn at the 50% probability level. H atoms and solvent molecules are omitted for clarity. Selected bond lengths (Å) and angles (deg.): C_1_–Si_1_ 1.851(8), Si_1_–Bi_1_ 2.576(2), C_1_⋯C_1a_ 2.268, Si_1_–Bi_1_–Si_1a_ 86.25(8), C_1_–Si_1_–Bi_1_ 117.1(3).

An scXRD analysis revealed that compound 2 crystallizes as a neutral Bi^I^ radical complex in the orthorhombic space group *Cmcm*. Its molecular structure features an open-cage *nido*-carborane backbone with a C⋯C distance of 2.268 Å (Fig. 3). The Si_1_–Bi_1_ bond length of 2.576(2) Å is comparable to those in complexes 1a and 1b (2.5774(6)–2.5958(9) Å). However, the Si_1_–C_1_ bond in complex 2 (1.851(8) Å) is significantly shorter than in 1a and 1b (1.923(2)–1.937(4) Å). Notably, the Si_1_–Bi_1_–Si_1a_ bond angle in complex 2 (86.25(8)°) is substantially larger than in 1a and 1b (∼79°), likely due to the open-cage nature of the carborane backbone.

**Fig. 4:**
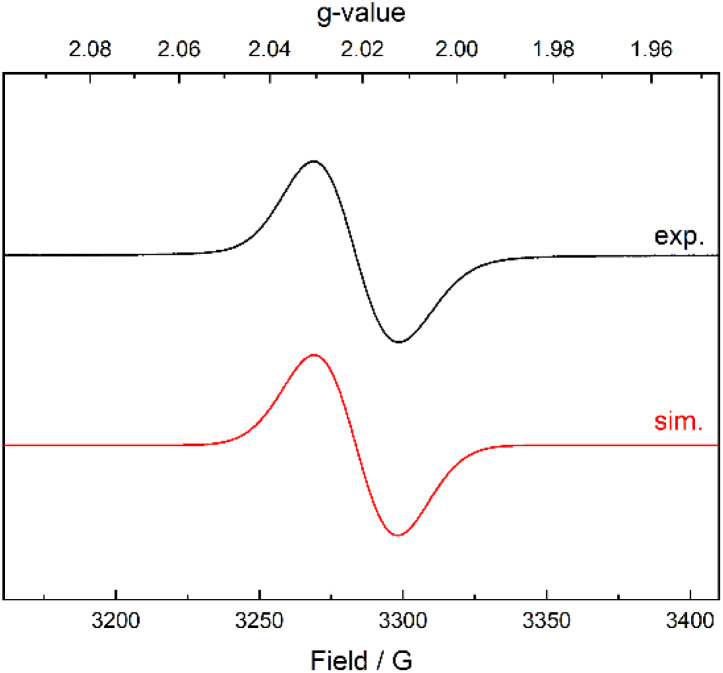
EPR spectrum of compound 2 in THF (top) recorded at 293 K and the corresponding simulation (bottom). The *g*-value of the radical species is 2.023 and the line width 29.1 G.

Compound 2 is paramagnetic and shows broad resonance peaks in solution ^1^H NMR spectra at room temperature (see ESI, Fig. S13†). Accordingly, the electron paramagnetic resonance (EPR) spectrum of 2 recorded at room temperature in THF (Fig. 4) exhibits an isotropic signal at *g* = 2.0229 (line width = 29.1 G). Though the band shape is very similar to the one of the known N^I^ anionic carborane radical {[Si^II^(*nido*-CB)Si^II^]N^I^},^35^ the *g*-value is slightly higher than those typical for such organic radicals. Magnetic interactions with the nearby very heavy Bi nucleus could be the origin. An unpaired spin located at the Bi itself can be excluded based on the EPR spectrum at 10 K (see ESI, Fig. S14†), which lacks the characteristic broad and multiline features of a Bi-centred radical (Bi^0^).^26^ Overall, the observations indicate that the unpaired electron is localized in the carborane cage (see electronic structure discussion below).

To investigate the reversibility of the latter one-electron reduction, compound 2 was allowed to react with equimolar amount of AgOTf in THF at room temperature. Indeed, compound 1b was quantitatively regenerated after stirring for 10 minutes (Scheme 2). Given the carborane moiety of bis(NHSi) can store one or two electrons, we further explored the two-electron reduction of 1b. The reaction of 1b with two molar equiv. of potassium naphthalenide (KC_10_H_8_) in THF at −30 °C yielded complex ({[Si^II^(*nido*-CB)Si^II^]Bi}K(thf)_2_)_2_ ([3K(thf)_2_]_2_) as a brown powder in 51% yield (Scheme 2). Its ^1^H NMR spectrum in THF-*d*_8_ displays one singlet at *δ* = 1.29 ppm for the *tert*-butyl groups, while the ^29^Si{^1^H} NMR spectrum exhibits a singlet at *δ* = 51.8 ppm, significantly upfield-shifted compared to 1a (*δ* = 68.7 ppm) and 1b (*δ* = 66.9 ppm).

**Fig. 5:**
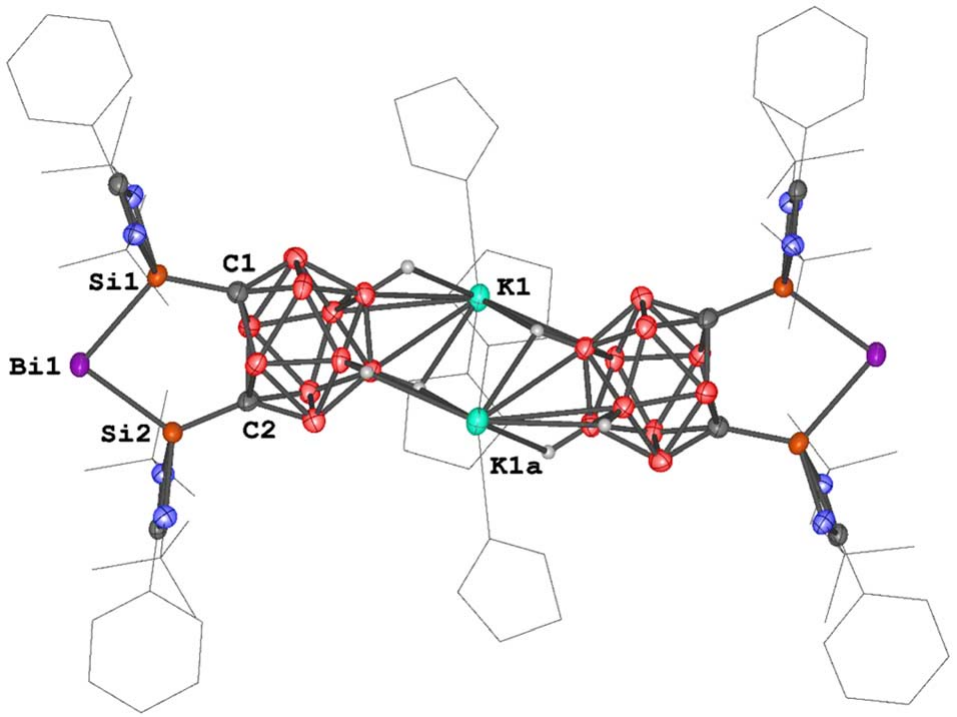
Molecular structure of ([3K(thf)_2_]_2_). Thermal ellipsoids are drawn at the 50% probability level. H atoms and solvent molecules are omitted for clarity. Selected bond lengths (Å) and angles (deg.): Bi_1_–Si_1_ 2.6266(13), Bi_1_–Si_2_ 2.6138(13), C_1_⋯C_2_ 2.576, Si_1_–C_1_ 1.786(5), Si_2_–C_2_ 1.789(5), Si_2_–Bi_1_–Si_1_ 90.27(4), C_1_–Si_1_–Bi_1_ 116.52(16), C_2_–Si_2_–Bi_1_ 115.93(16).

An scXRD analysis reveals that ([3K(thf)_2_]_2_) adopts a dimeric structure in the solid state, with two [K(thf)_2_]^+^ moieties acting as linkers *via* B–H⋯K⋯H–B interactions (Fig. 5). The Si–Bi bond lengths in ([3K(thf)_2_]_2_) (2.6266(13) and 2.6138(13) Å) are slightly longer than those in 1a and 1b (2.5774(6)–2.5958(9) Å) and in 2 (2.576(2) Å). Additionally, the Si–Bi_1_–Si bond angle in ([3K(thf)_2_]_2_) (90.27(4)°) is larger than in 1a and 1b (∼79°) and 2 (86.25(8)°). In line with that, the carborane backbone in ([3K(thf)_2_]_2_) features a C⋯C distance of 2.576 Å, notably longer than that in 2 (2.268 Å), indicating a further reduced carborane cage. Furthermore, the Si–C distances in ([3K(thf)_2_]_2_) (1.786(5) and 1.789(5) Å) are significantly shorter than those in 1a and 1b (1.923(2)–1.937(4) Å) and in 2 (1.851(8) Å). These structural features confirm that the open cage in ([3K(thf)_2_]_2_) corresponds to a *nido*-carborane anion. It should be mentioned that complex 2 can also be synthesized *via* a metathesis reaction between 1b and ([3K(thf)_2_]_2_) in THF at room temperature. In addition, complex ([3K(thf)_2_]_2_) can be obtained through the one-electron reduction of complex 2 with KC_8_ in THF (Scheme 2). This indirectly confirms the redox reversibility between the family members of this Bi^I^ series.

**Scheme 3:**
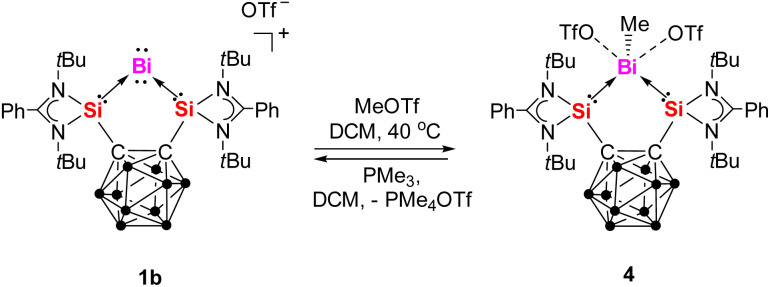
Reaction of 1b with MeOTf and the reverse reaction with PMe_3_.

The reactivity of 1b towards methyl trifluoromethanesulfonate (MeOTf) was also investigated to evaluate the nucleophilic character of Bi^I^. Upon addition of MeOTf at 40 °C in DCM, the yellow solution of 1b gradually decolorized over 4 hours, yielding colorless {[Si^II^(*closo*-CB)Si^II^]BiMe} [OTf]_2_ (4) in 61% yield after workup (Scheme 3). The ^1^H NMR spectrum of 4 in DCM-*d*_2_ displays a singlet at *δ* = 2.47 ppm for the methyl group,^37^ downfield-shifted compared to free BiMe_3_ (*δ* = 1.11 ppm).^37*a*^ The ^29^Si{^1^H} NMR spectrum exhibits a singlet at *δ* = 62.0 ppm, significantly upfield-shifted compared to 1a (*δ* = 68.7 ppm) and 1b (*δ* = 66.9 ppm) but downfield-shifted relative to ([3K(thf)_2_]_2_) (*δ* = 51.8 ppm). The ^19^F NMR spectrum shows a singlet at *δ* = −78.7 ppm. Notably, treatment of 4 with PMe_3_ in DCM at room temperature quantitatively regenerated 1b immediately along with the formation of PMe_4_OTf.

**Fig. 6:**
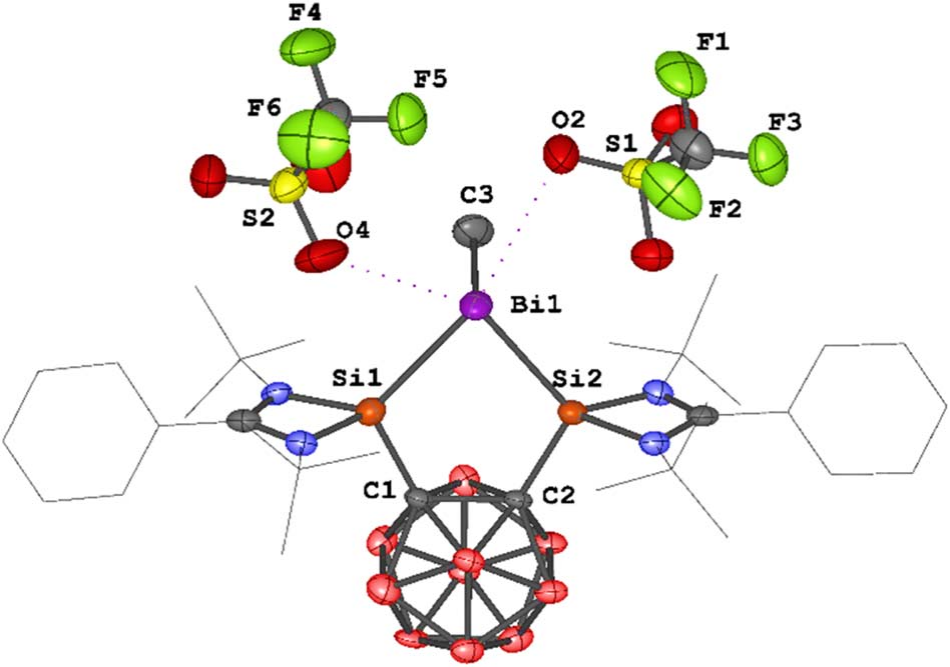
Molecular structure of 4. Thermal ellipsoids are drawn at the 50% probability level. H atoms and solvent molecules are omitted for clarity. Selected bond lengths (Å) and angles (deg.): Bi_1_–Si_2_ 2.7003(17), Bi_1_–Si_1_ 2.7160(19), Bi_1_–C_3_ 2.278(9), C_2_–C_1_ 1.717(9), Si_1_–C_1_ 1.929(7), Si_2_–C_2_ 1.917(6), Si_1_–Bi_1_–Si_2_ 80.44(5), C_1_–Si_1_–Bi_1_ 112.1(2), C_2_–Si_2_–Bi_1_ 112.24(19), C_3_–Bi_1_–Si_1_ 96.5(2), C_3_–Bi_1_–Si_2_ 94.7(3).

An scXRD analysis revealed that 4 crystallizes in the monoclinic space group *P*2_1_/*n*. The dication in 4 features a five-membered C_2_Si_2_Bi ring, with a methyl group and two triflate anions coordinated to the bismuth atom (Fig. 6). The bismuth center thus adopts a distorted tetragonal pyramidal geometry (*τ*_5_ = 0.25),^37*b*^ with the methyl group occupying the apical position, suggesting the presence of a lone electron pair opposite the tetragonal plane. The Si–Bi bond lengths in 4 (2.7003(17) and 2.7160(19) Å) are significantly longer than those observed in complexes 1–3 (1: 2.5774(6)–2.5958(9) Å; 2: 2.576(2) Å; 3: 2.6266(13) and 2.6138(13) Å). The Bi_1_–C_3_ bond length of 2.278(9) Å in 4 is comparable to that in the [BiMe]^2+^ complexes derived from II (2.300 Å)^25^ and III (2.247(7) Å).^26^ Notably, the shortest Bi⋯O interaction between the bismuth center and the two triflate anions in 4 is 3.025 Å, indicating weak coordination between the bismuth center and the triflate anions.

### Computational analysis

To elucidate the electronic structures of these monovalent bismuth complexes, Density Functional Theory (DFT) calculations were performed using the CAM-B3LYP/def2-TZVPD level in Gaussian 16 (see ESI† for details).^38^Table 1 compiles the HOMO–LUMO gaps of these species, confirming the predominantly single-determinant character of their wavefunctions and thus supporting the use of Kohn–Sham DFT as an appropriate method. Moreover, to discard large electron delocalization errors, we have compared CAM-B3LYP and B3LYP values, the latter of which are included in the ESI for comparison (see ESI, Table S11 and Fig. S34†). The experimental scXRD data were used for the analysis, although several optimized structures—which show good agreement with the experimental ones—are also included in the ESI for comparison (see Fig. S35 and S36†). Table 1 includes the ionization potential of these species, which serves as an indication of their stability with respect to the loss of one electron, and the first hyperpolarizabilities, *β*. The first hyperpolarizability increases from 1a to 2, and 2 to 3, reaching relatively large values which are compatible with delocalized charge distributions. In order to assess the changes in the electron distribution upon reduction, we have also employed the quantum theory of atoms in molecules (QTAIM)^39^ partition to compute atomic charges and delocalization indices (DI), which are covalent bond orders.^40,41^ We also run various tests to discard the electride character of 2 and 3.^42,43^ We carried out a complete topological analysis of the electron density for 1a, 2, and 3, identified the critical points according to QTAIM, and examined the regions with negative values of the Laplacian of the electron density. No features indicative of an isolated electron—such as a non-nuclear attractor or regions of localized electron density revealed by Laplacian analysis, which are characteristic of electrides—were identified (see Section D4 in the ESI†). Conversely, the first hyperpolarizabilities of these compounds (see Table 1) are consistent with the presence of labile and loosely bound electrons, albeit not isolated or localized as expected in electrides.

**Table 1:** Molecular properties (ionization potential (IP), HOMO–LUMO gap (H–L gap), first hyperpolarizabilities, *β*, and QTAIM charges) computed for the XRD structures of 1a, 2, and 3 at the CAM-B3LYP/def2-TZVPD level of theory. Charges of C_1_ and C_2_ are considered equivalent. For more details see Fig. S31. *β* values are expressed in (a.u.)

QTAIM charges	1a	2	3
Bi	0.10	−0.31	−0.45
C	−1.75	−2.28	−2.54
BH cage	1.98	2.28	1.99
IP (eV)	9.10^a^	4.59	2.58
H–L gap (eV)	6.02^a^	4.23	3.15
*β* (a.u.)	8.40 × 10^2,^^a^	2.74 × 10^3^	7.18 × 10^3^

a Values computed without considering the iodide counterion to permit a direct comparison among the species.

**Fig. 7:**
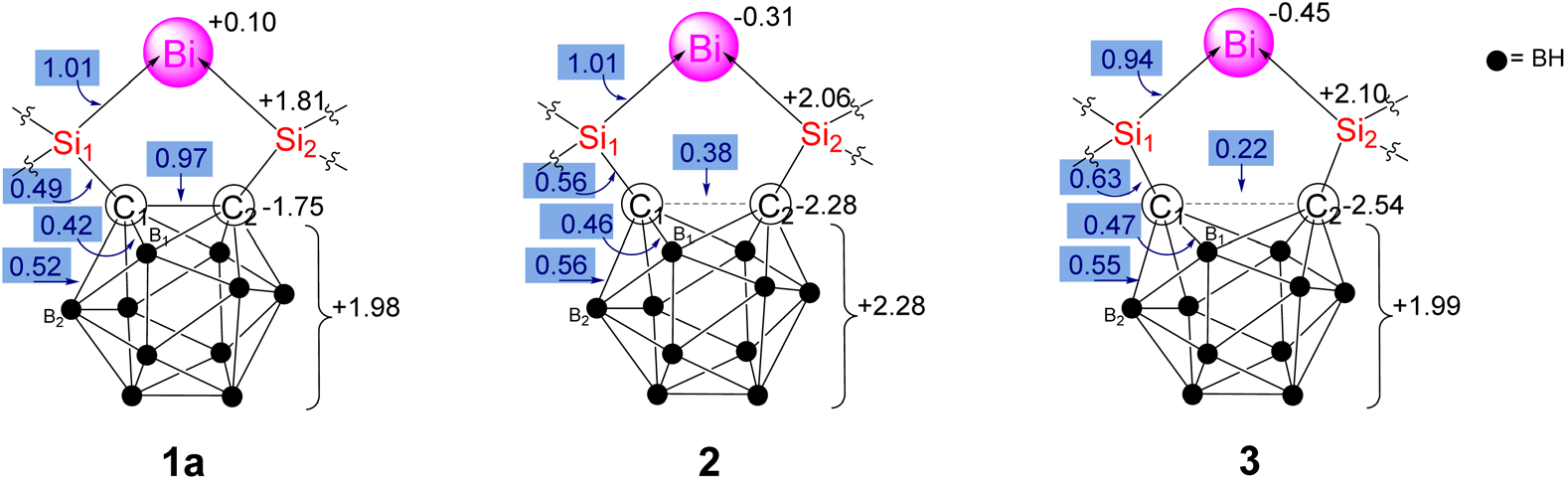
Evolution of selected QTAIM atomic charges (in black) and delocalization indices (shaded and blue) of 1a, 2 and 3 structures upon reduction.

The skeleton of 1a, 2, and 3 is divided into four parts: the Bi center, the two amidinato silylene units, and the carborane cage. The amidinato silylene units barely change upon reduction from 1a to 2, and 2 to 3, and their data is only included in the ESI† for the sake of completion. Fig. 7 and Table 1 contain data concerning the electron distribution of the relevant parts of structures 1a, 2, and 3.

We focus on the Bi center, the two carbon atoms in the carborane cage, and the rest of the cage (BH cage, hereafter). 1a exhibits a regular 2c–2e bond (DI = 1.01) between Bi and Si (which is essentially maintained after reduction to 2 and 3), while the bonds between Si and the C atoms in the carborane cage are partially covalent (DI = 0.49) with a high ionic component (Si holds a +2.06 charge, whereas C has a large negative charge of −1.75*e*). 1a also exhibits a C–C covalent bond (DI = 0.97) within the carborane cage.

Upon reduction of 1a to 2, the most significant change is the reduction of the C–C bonding interaction (DI = 0.38), which further decreases upon reduction to 3 (DI = 0.22). This C–C weakening is caused by the opening of the cage at the top, as reflected in the increase of the C–C bond distance from 1.69 Å in 1a to 2.27 Å in 2, and subsequently to 2.58 Å in 3. The cage opening is accompanied by a slight increase of the covalent bond orders between C and the neighboring B atoms (see ESI, Fig. S31†), and a slight increase of the Si–C bond strength, which is reflected by the higher covalent character (DI = 0.56 in 2, DI = 0.63 in 3). This electron reorganization is also reflected in the picture of the Laplacian of the electron density given in Fig. S32 and S33,† and the number of electrons localized in the C atoms (see ESI, Fig. S31†).

Hence, upon reduction from 1a to 2, the extra electron mostly localizes in the carborane cage, especially in the carbon atoms, as the BH cage actually loses some electron density upon reduction. The Bi atomic charge also increases by 0.4*e*. Based on the calculated partial charge distribution, resonance structure 2′ (Scheme 2) is proposed for compound 2, in which the negative charges are delocalized over the bismuth atom and the carborane cage, while the two positive charges are distributed over the two silicon atoms. Upon reduction from 2 to 3, 0.26 additional electrons localize in each C atom, whereas the other half electron is split between the Bi, which has now −0.45 electrons, and the BH cage, which restores the +2 charge it had in 1a.

**Fig. 8:**
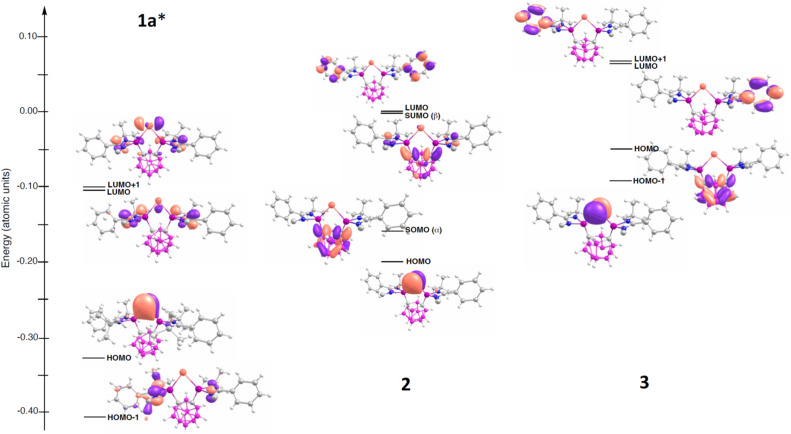
Molecular orbital diagrams for selected orbitals of complexes 1a (*without considering the I^−^ counterion), 2, and 3. The front *tert*-butyl groups of the silylenyl units are removed for clarity. Molecular orbitals isovalue of 0.050 e Å–3.

The analysis of the molecular orbitals aligns well with the conclusions drawn thus far (see Fig. 8). The SOMO of the Bi^I^ compound 2 shows a large fraction of the electron density of the unpaired electron on the C atoms of the carborane (Fig. 8). This stabilized σ* orbital is reminiscent of the N^I^ radical {[Si^II^(*closo*-CB)Si^II^]N^I^} supported by the same bis(silylenyl) carborane ligand^35^ and the diphenyl-o-carborane system reported by Adillon *et al.*^44^

## Conclusion

In summary, we have successfully synthesized and characterized a series of bis(silylene)-stabilized monovalent single atom bismuth complexes supported by a chelating bis(silylenyl)-*o*-carborane ligand. The redox-active nature of the carborane scaffold plays a crucial role in modulating the electronic structures of these Bi^I^ complexes. The one-electron reduction of the cationic complex 1b with KC_8_ afforded the neutral radical complex 2, while its two-electron reduction with KC_10_H_8_ led to the formation of the anionic dimeric complex 3. These transformations highlight the remarkable redox flexibility of the bis(silylenyl)carborane framework in stabilizing monovalent bismuth. Furthermore, the reversibility of the redox processes, as demonstrated by the quantitative regeneration of 1b upon oxidation of 2 with AgOTf, underscores the dynamic redox behavior of these complexes. X-ray crystallographic analyses and spectroscopic data reveal significant structural variations among 1–3, particularly in the Bi–Si bonding and carborane core geometry. DFT calculations revealed that upon reduction from 1a to 2, the added electron predominantly localizes within the carborane cage, with a marked preference for the carbon atoms. This localization weakens the central C–C bond. A similar trend is observed in the subsequent reduction from 2 to 3, where the additional electron again accumulates in the carborane framework. Further analysis rules out the possibility that these species exhibit the characteristics of molecular electrides. Overall, this work expands the understanding of bismuth redox chemistry and the role of cooperative carborane-based silylene ligands in stabilizing low-valent main-group species. The unique electronic properties of these robust Bi^I^ complexes are expected to pave the way to new types of Bi-based redox catalysts.

## Author contributions

Jian Xu carried out the synthetic experiments and analyzed the experimental data. Shenglai Yao assisted in the XRD refinement of the compounds and edited the manuscript. Christian Lorent collected the EPR data. Verònica Postils performed the DFT calculations. Verònica Postils and Eduard Matito analyzed the electronic structure of the compounds. Matthias Driess supervised the work and edited the manuscript. The manuscript was written through the contribution of all authors.

## Conflicts of interest

There are no conflicts to declare.

## Supplementary Material

SC-016-D5SC02644J-s001

SC-016-D5SC02644J-s002

## Data Availability

All experimental and computational data associated with this work are available in the ESI.†
