# Supplementary material for: Isolable monoatomic monovalent bismuth complexes with a redox non-innocent bis-silylenyl carborane ligand
Source: Chem Sci. 2025 May 19;16(24):10826–32. doi: 10.1039/d5sc02644j (PMC12100516; doi:10.1039/d5sc02644j)
Supplement: SC-016-D5SC02644J-s001 [file SC-016-D5SC02644J-s001.pdf]

Supplementary Information (SI) for Chemical Science.
This journal is © The Royal Society of Chemistry 2025

Supporting Information

Isolable Monoatomic Monovalent Bismuth Complexes with a Redox Non-Innocent Bis-silylenyl Carborane Ligand

Jian Xu,[a] Shenglai Yao,[a] Verònica Postils,[b] Eduard Matito,[c,d] Christian Lorent,[e] and Matthias Driess*[a]

a. *Metalorganic and Inorganic Materials, Department of Chemistry, Technische Universität Berlin, 10623 Berlin, Germany;*

b. *Theoretical Chemistry Group, Molecular Chemistry, Materials and Catalysis Division (MOST), Institut of Condensed Matter and Nanosciences, Université Catholique de Louvain, Place Louis Pasteur 1, B-1348 Louvain-la-Neuve, Belgium;*

c. *Donostia International Physics Center (DIPC)*, 20018 Donostia, Euskadi, Spain;

d. *Ikerbasque Foundation for Science, Plaza Euskadi 5, 48009 Bilbao, Euskadi, Spain;*

e. *Physical and Biophysical Chemistry, Department of Chemistry Technische Universität Berlin, 10623 Berlin, Germany.*

Content

A. Experimental Procedures 2
A1. General Considerations 2
A2. Single-Crystal X-ray Structure Determination 2
A3. Cyclic Voltammetry Measurement 3
A4. EPR Spectroscopy 3
B. Synthesis and Characterization 4
C. X-ray Crystallographic Data 24
D. Theoretical Calculations 34
D5. Cartesian Coordinates in Angstrom 40
References 53

A. Experimental Procedures

A1. General Considerations

All experiments were carried out under dry oxygen-free nitrogen using standard Schlenk techniques or MBraun glove box fitted with a gas purification and recirculation unit. Solvents were dried by standard methods and freshly distilled before use. The bis(silylenyl)-*o*-carborane [Si^{II}(*closo*-CB)Si^{II}] (**A**, Si^{II} = PhC(N*t*Bu)$_2$Si, CB = *o*-$C_2B_{10}H_{10}$)[1] was synthesized according to reported procedures. The solution NMR spectra were recorded on Bruker Spectrometers AV 400 and 500 with residual solvent signals as internal reference (^{1}H NMR: THF-d_8, 3.58 and 1.72 ppm, DCM-d_2, 5.32 ppm; ^{13}C{^{1}H} NMR: THF-d_8: 67.21 and 25.31 ppm, DCM-d_2, 53.84 ppm) and external standards (^{11}B{^{1}H} NMR and ^{19}F{^{1}H} NMR: BF_3.Et_2O). The following abbreviations were used to describe peak patterns when appropriate: br = broad, s = singlet, d = doublet, t = triplet, dd = doublet of doublets, m = multiplet. Elemental analyses were performed by the analytical labor service at the Institute of Chemistry, Technical University of Berlin, Germany. High-resolution ESI-MS were measured on a Thermo Scientific LTQ orbitrap XL. UV/Vis spectra were recorded on an Analytik Jena Specord S600 diode array spectrometer.

A2. Single-Crystal X-ray Structure Determination

Crystals were each mounted on a glass capillary in perfluorinated oil and measured in a cold N_2 flow. The data of all compounds were collected on an Oxford Diffraction SuperNova, Single source at offset, Atlas at 110K (Cu-Kα radiation, λ = 1.54184 Å). The structures were solved by direct methods and refined on F^2 with the SHELX-2014 and Olex2 software packages.[2,3]

In the molecular structures of compounds **1a** and **2**, strongly disordered solvent molecules C_6H_6 (**1a**) and C_7H_8 (**2**) were treated using Solvent Masking in Olex2. In the molecular structure of compound **2**, the Bi1 atom is disordered over two positions with an approximate occupancy ratio of 0.96:0.04 (Bi1: Bi1a). In the molecular structure of compound **3**, one of the solvent THF is disordered over two positions (part 1: O3, C41 and C42 atom; part 2: O3a, C41a and C42a atom) with an approximate occupancy ratio of 0.77: 0.23 (part 1: part 2). In the molecular structure of compound **4**, residual electron density was observed close to the center of the bismuth, which may be due to anharmonic displacement of the heavy metal atoms and

the Bi1 atom is also disordered over two positions with an approximate occupancy ratio of 0.96:0.04 (Bi1: Bi1a).

CCDC: 2428091 (**1a**), 2428092 (**1b**), 2428093 (**2**), 2428094 (**3**) and 2428095 (**4**), contain the supplementary crystallographic data for this paper. These data can be obtained free of charge from The Cambridge Crystallographic Data Centre via www.ccdc.cam.ac.uk/structures/

A3. Cyclic Voltammetry Measurement

Cyclic voltammetry (CV) measurements of compound **1b** were performed in a standard three-electrode electrochemical cell having Pt-wire used as an auxiliary electrode, glassy carbon (3 mm diameter) as working electrode and Pt-wire as a pseudo reference electrode at 295 K using a Biologic SP-150 potentiostat. All cyclic voltammograms were referenced against the Cp_2Fe/Cp_2Fe^+ redox couple (Fc/Fc^+), which was used as an internal standard. 0.1 M tetrabutyl ammonium hexafluorophosphate ($TBAPF_6$) in THF was used as an electrolyte. The iR-drop was determined and compensated by using the impedance measurement technique implemented in the EC-Lab Software V10.37.

A4. EPR Spectroscopy

EPR data was measured with a Bruker EMXplus spectrometer using an ER 4122 SHQE resonator. An Oxford EPR 900 helium flow cryostat and Oxford ITC4 temperature controller was used for accumulating spectra at cryogenic conditions. Experimental conditions: 1 mW microwave power, microwave frequency: 9.3 GHz, 1 G modulation amplitude, 100 kHz modulation frequency. Numerical simulation of the EPR spectrum was performed using the Matlab toolbox Easyspin 5.2.36.[4]

B. Synthesis and Characterization

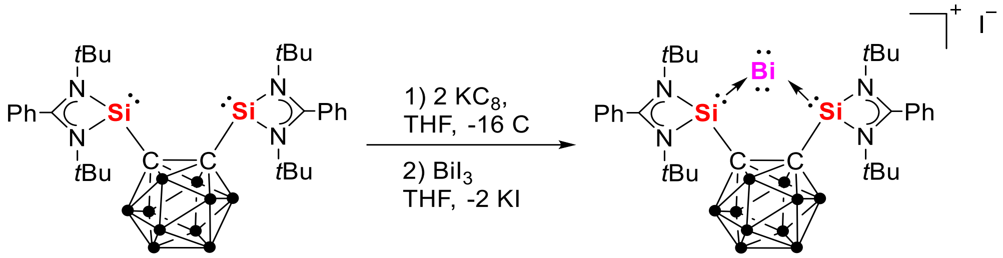

A **1a**

Synthesis of compound 1. To a Schlenk flask charged with **A** (330 mg, 0.50 mmol) and potassium graphite KC_8 (140 mg, 1.04 mmol) was added 40 mL THF at room temperature with stirring. After stirring at room temperature for 2.5 h, the resulting suspension was filtered to give an orange solution. The latter solution was then cooled to -30 °C and BiI_3 (290 mg, 0.50 mmol) in 20 mL THF was added with stirring. The reaction mixture was allowed to warm to room temperature and stirred further for 4 h. All volatiles were then removed under vacuum and the residue was extracted with DCM (2 × 20 mL). The resulting suspension was filtered to give an orange solution and all volatiles were then removed under vacuum again to obtain the crude product. Recrystallization with THF furnished pure **1a** as yellow needle crystals. (285 mg, isolated yield 57 %). Orange needle crystals suitable for X-ray diffraction analysis were obtained from a benzene solution at room temperature.

M.p. 190.5 ^{o}C (decomp.).

^{1}H NMR (400 MHz, DCM-d_2) δ/ppm = 7.83 (d, J = 7.0 Hz, 2H, Ar-H), 7.64 (m, 4H, Ar-H), 7.53 (t, J = 8.1 Hz, 2H, Ar-H), 7.39 (d, J = 7.7 Hz, 2H, Ar-H), 1.35 (s, 36H, C(CH_3)$_3$). The 10 BH signals are very broad and unresolved.

^{13}C{^{1}H} NMR (101 MHz, DCM-d_2) δ/ppm = 181.62 (s, NCN), 132.03, 131.43, 130.16, 128.99, 128.68 (s, Ar-C), 86.31 (s, carborane-C), 57.72 (s, NC(CH$_3$)$_3$), 32.63 (s, NC(CH$_3$)$_3$).

^{29}Si{^{1}H} NMR (79 MHz, DCM-d_2) δ/ppm = 68.74 (s).

^{11}B{^{1}H} NMR (160 MHz, DCM-d_2) δ/ppm = -13 – 0 (m, vb).

HR-MS (ESI): (m/z) calcd for $[M-I]^+$ ($C_{32}H_{56}B_{10}N_4Si_2Bi^+$): 870.4845; found: 870.4835.

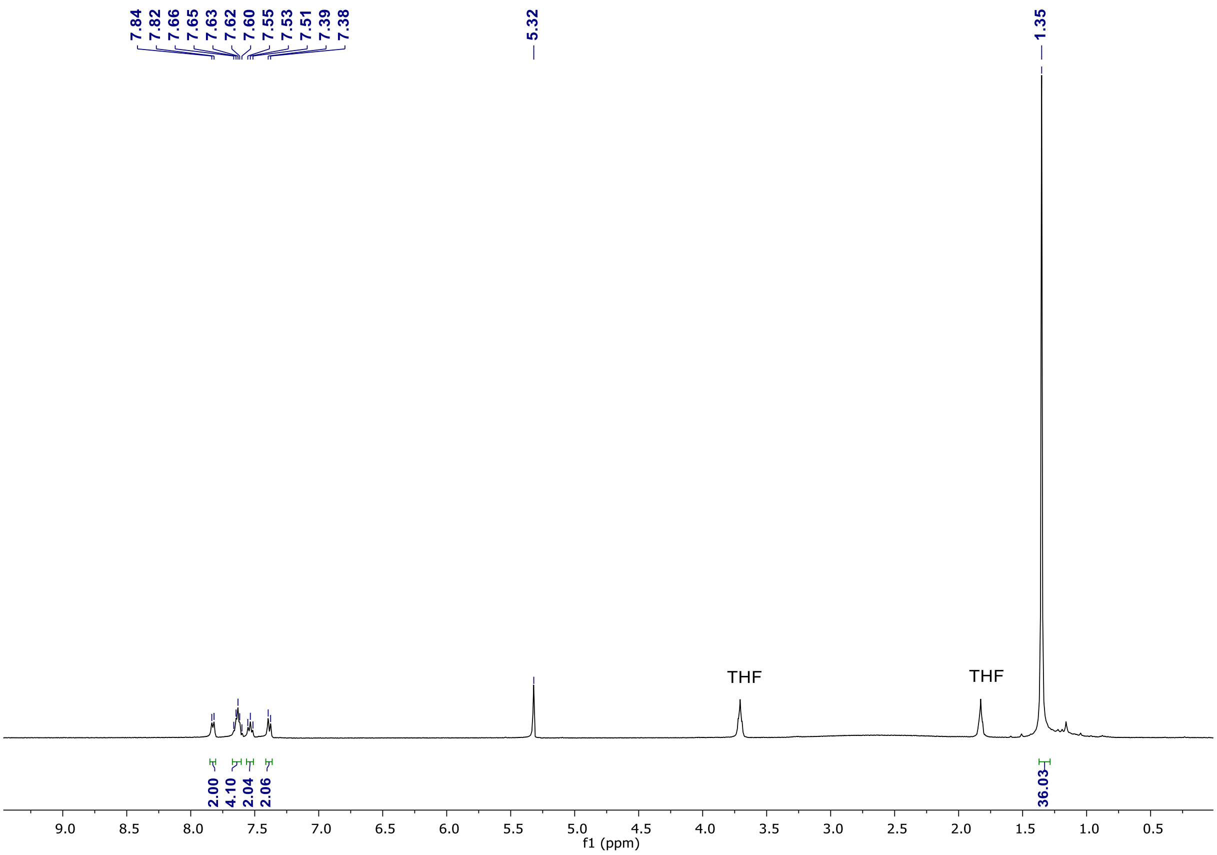

Figure S1. ^{1}H NMR spectrum of **1a** in DCM-d_2.

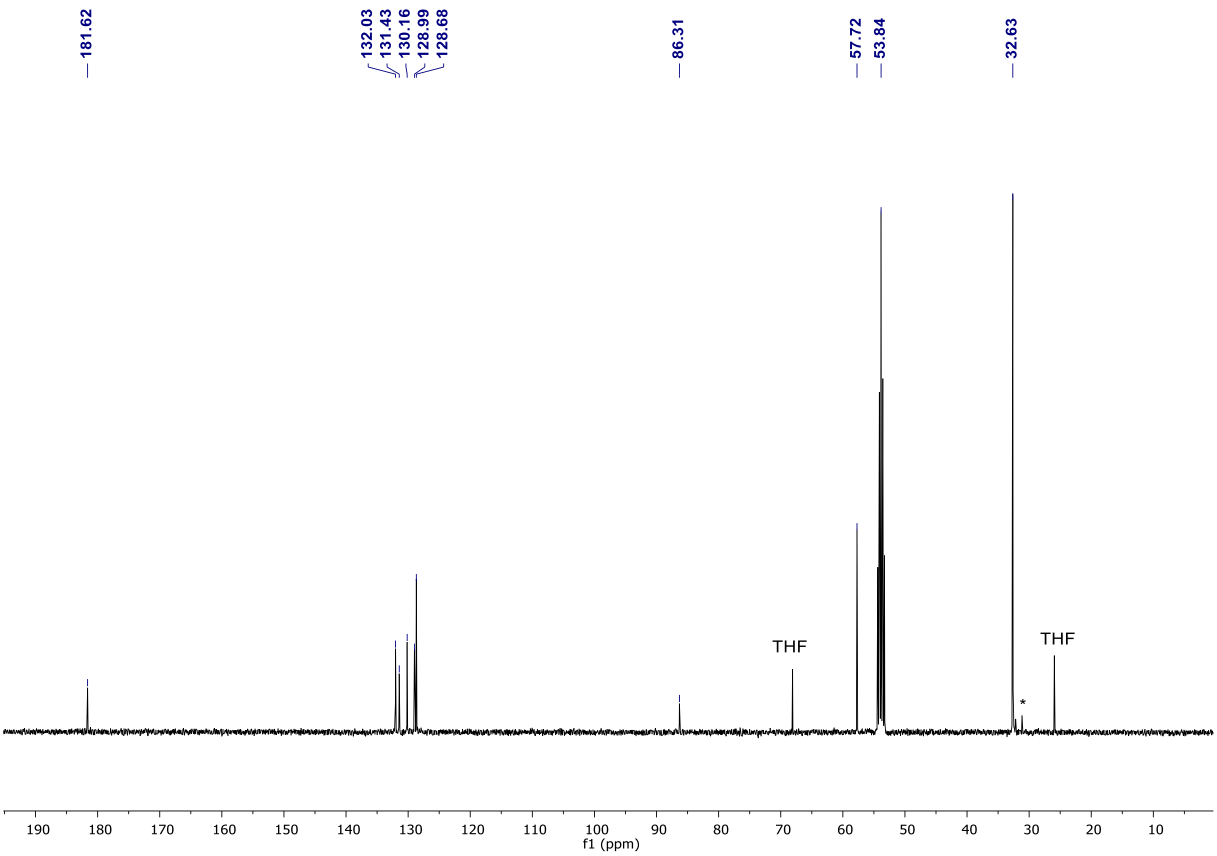

Figure S2. $^{13}C\{^{1}H\}$ NMR spectrum of **1a** in DCM-d_2. *Unidentified impurities.

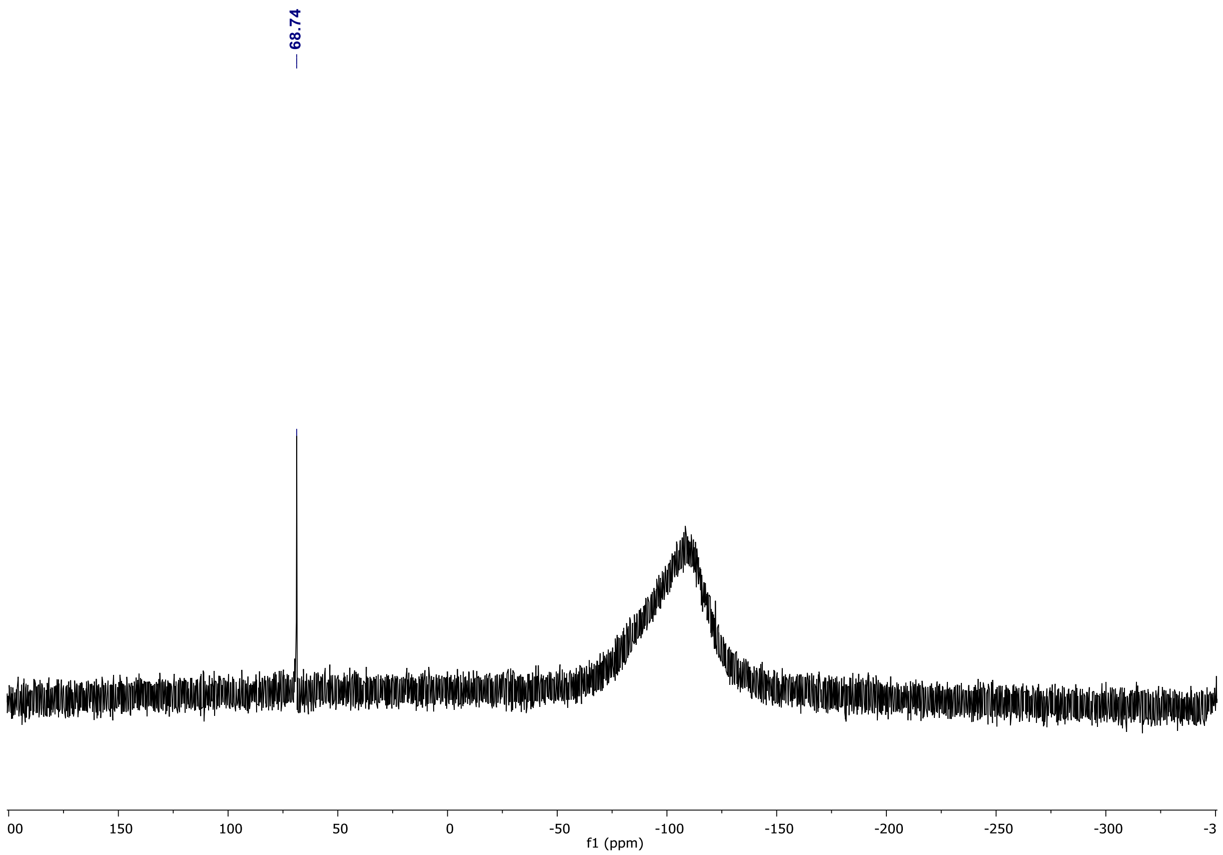

Figure S3. $^{29}Si\{^{1}H\}$ NMR spectrum of **1a** in DCM-d_2.

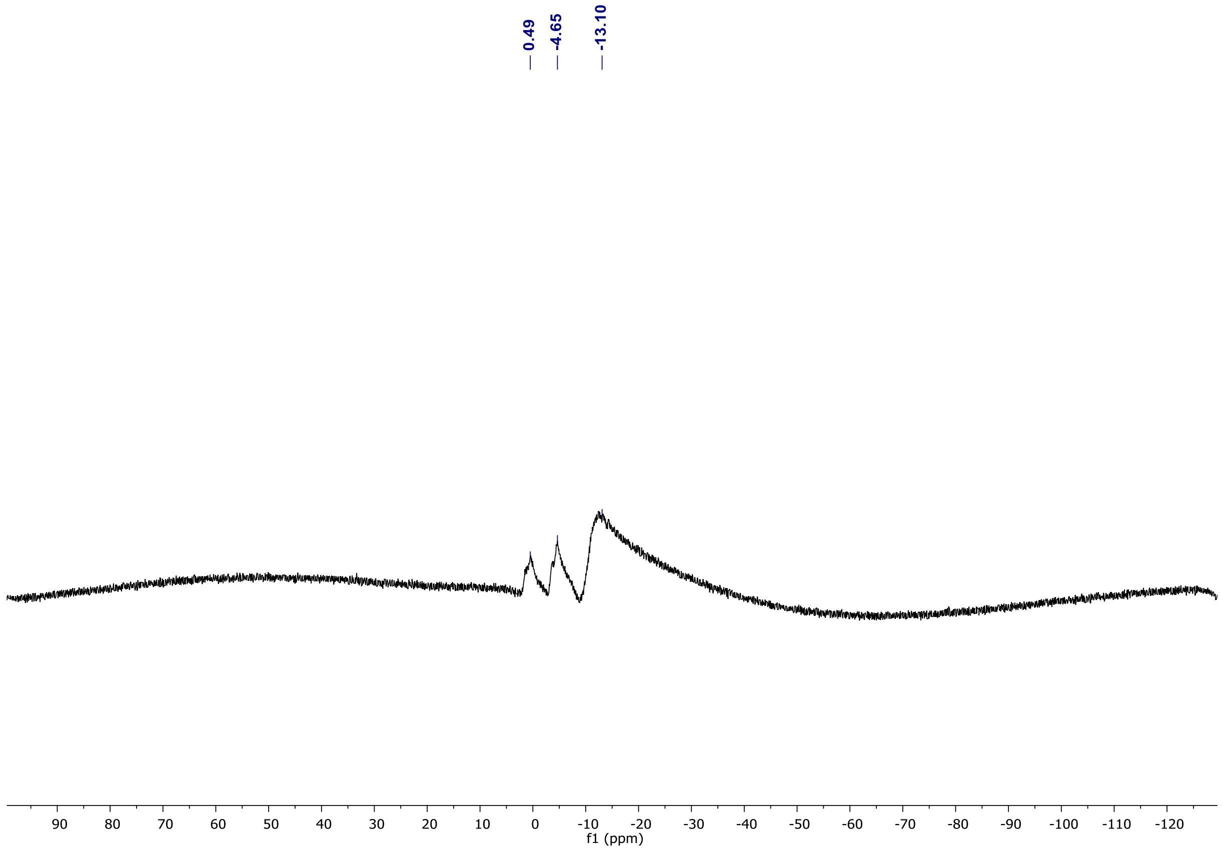

Figure S4. $^{11}B\{^{1}H\}$ NMR spectrum of **1a** in DCM-d_2.

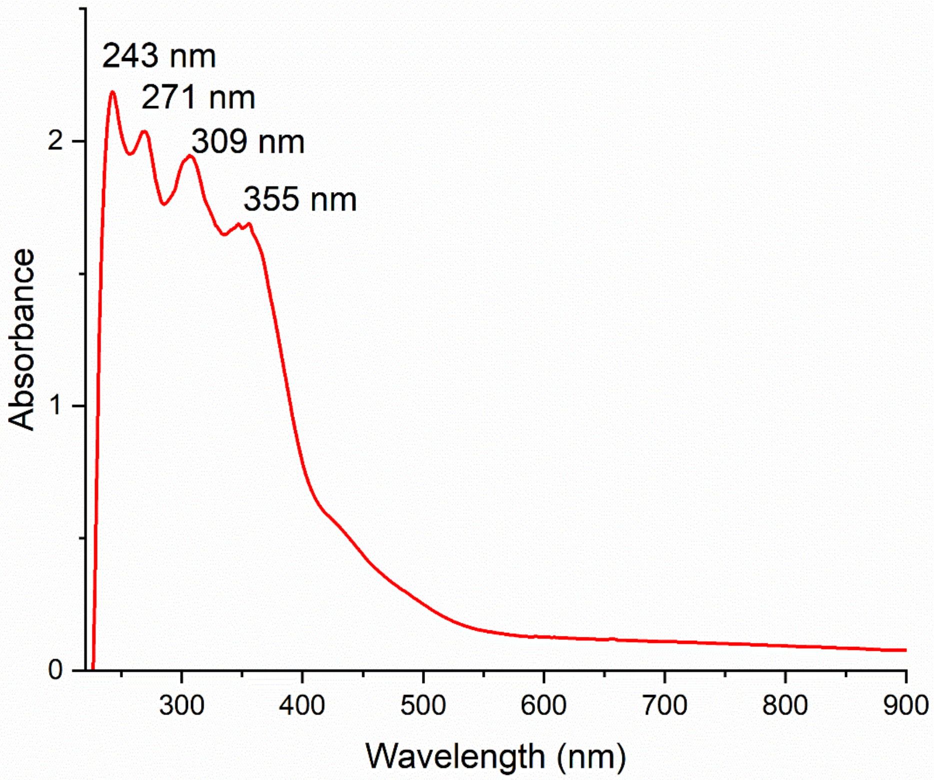

Figure S5. UV-Vis spectrum of **1a** in DCM.

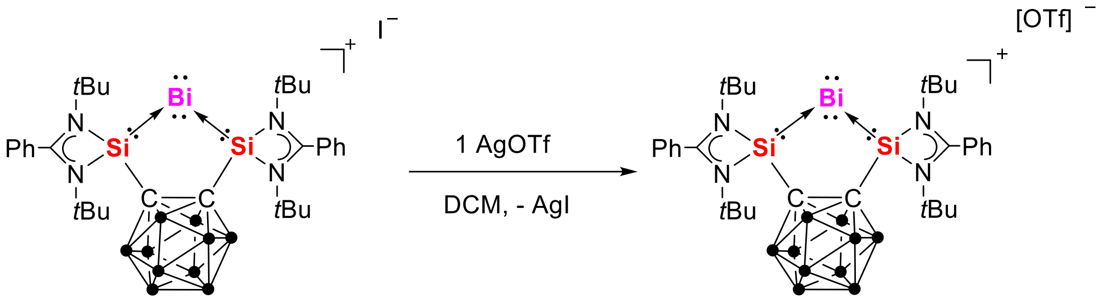

1a **1b**

Synthesis of compound 1b. To a mixture of compound **1a** (998 mg, 1 mmol) and AgOTf (257 mg, 1 mmol) in a 50 mL Schlenk flask was added 30 mL DCM at room temperature under stirring. The color of the mixture changed to deep orange immediately. After stirring for 30 min, the mixture was filtered and all volatiles were removed and dried under vacuum to afford compound **1b** as an orange powder. Recrystallization with Et_2O/DCM furnished pure **1b** as crystals at -20 °C (785 mg, 77% isolated yield). Yellow block crystals suitable for X-ray diffraction analysis were obtained from an Et_2O/DCM solution at 4 °C.

M.p. 208.5 °C (decomp.).

^{1}H NMR (500 MHz, DCM-d_2) δ/ppm = 7.71 – 7.62 (m, 4H, Ar-*H*), 7.62 – 7.56 (m, 4H, Ar-*H*), 7.40 (d, *J* = 7.8 Hz, 2H, Ar-*H*), 1.32 (s, 36H, C(CH_3)$_3$). The 10 B*H* signals are very broad and unresolved.

^{13}C{^{1}H} NMR (101 MHz, DCM-d_2) δ/ppm = 182.83 (s, N*C*N), 132.42, 130.89, 130.15, 129.25, 129.02, 127.79 (s, Ar-*C*), 121.29 (q, *J* = 320.9 Hz, *C*F_3), 85.45 (s, carborane-*C*), 58.08 (s, N*C*(CH_3)$_3$), 32.39 (s, NC(*C*H_3)$_3$).

^{29}Si{^{1}H} NMR (79 MHz, DCM-d_2) δ/ppm = 66.89 (s).

^{19}F NMR (471 MHz, DCM-d_2) δ/ppm = -78.67 (s).

^{11}B{^{1}H} NMR (160 MHz, DCM-d_2) δ/ppm = -11.02 – 1.64 (m, vb).

HR-MS (ESI): (m/z) calcd for [M-OTf]$^+$ ($C_{32}H_{56}B_{10}N_4Si_2Bi^+$): 870.4845; found: 870.4831.

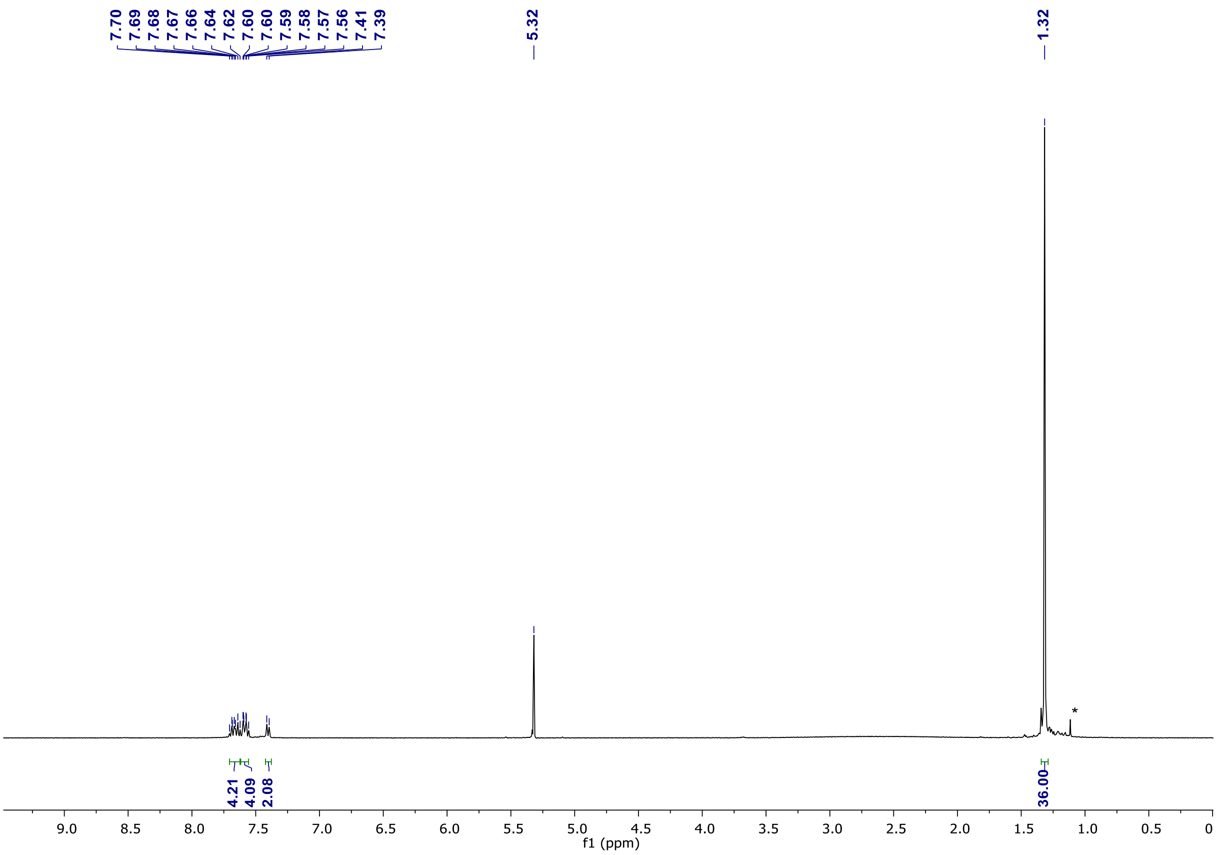

Figure S6. ^{1}H NMR spectrum of **1b** in DCM-d_2. *Unidentified impurities.

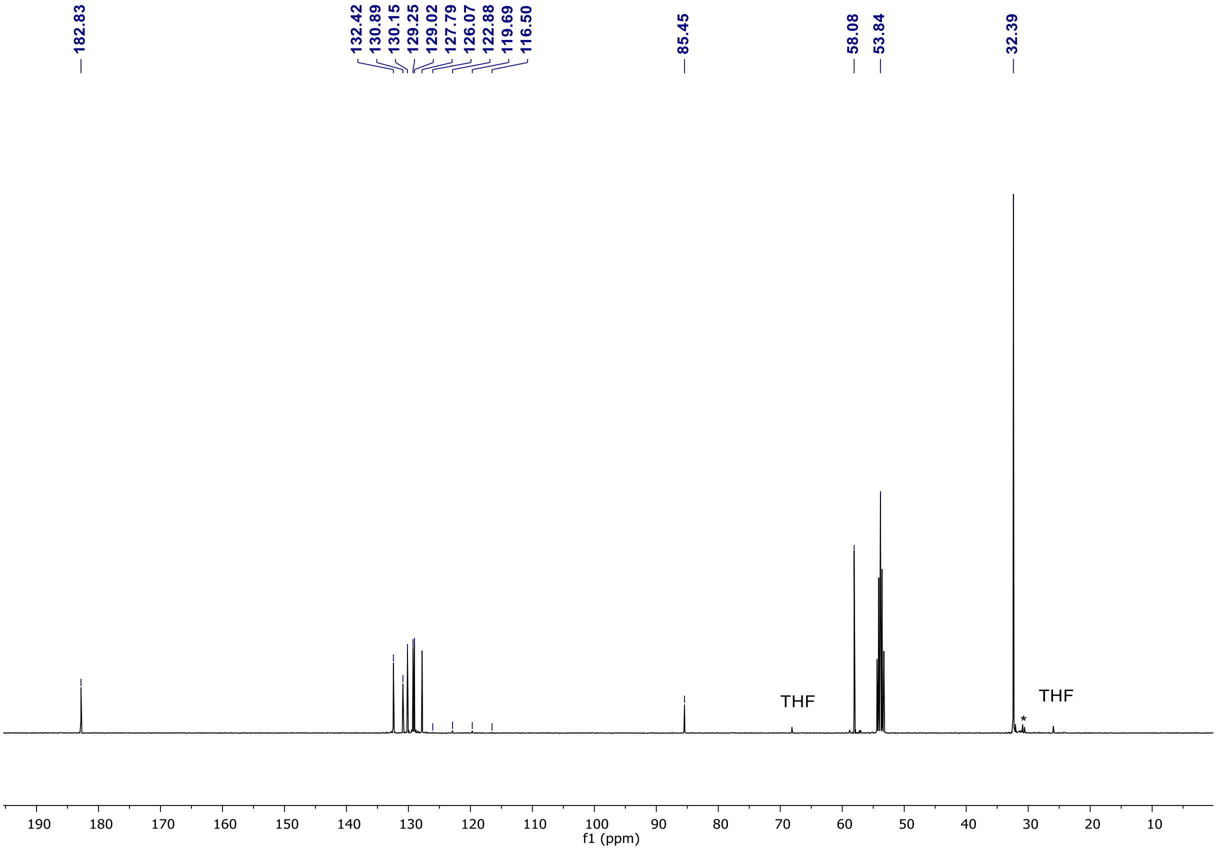

Figure S7. $^{13}C\{^{1}H\}$ NMR spectrum of **1b** in DCM-d_2. *Unidentified impurities.

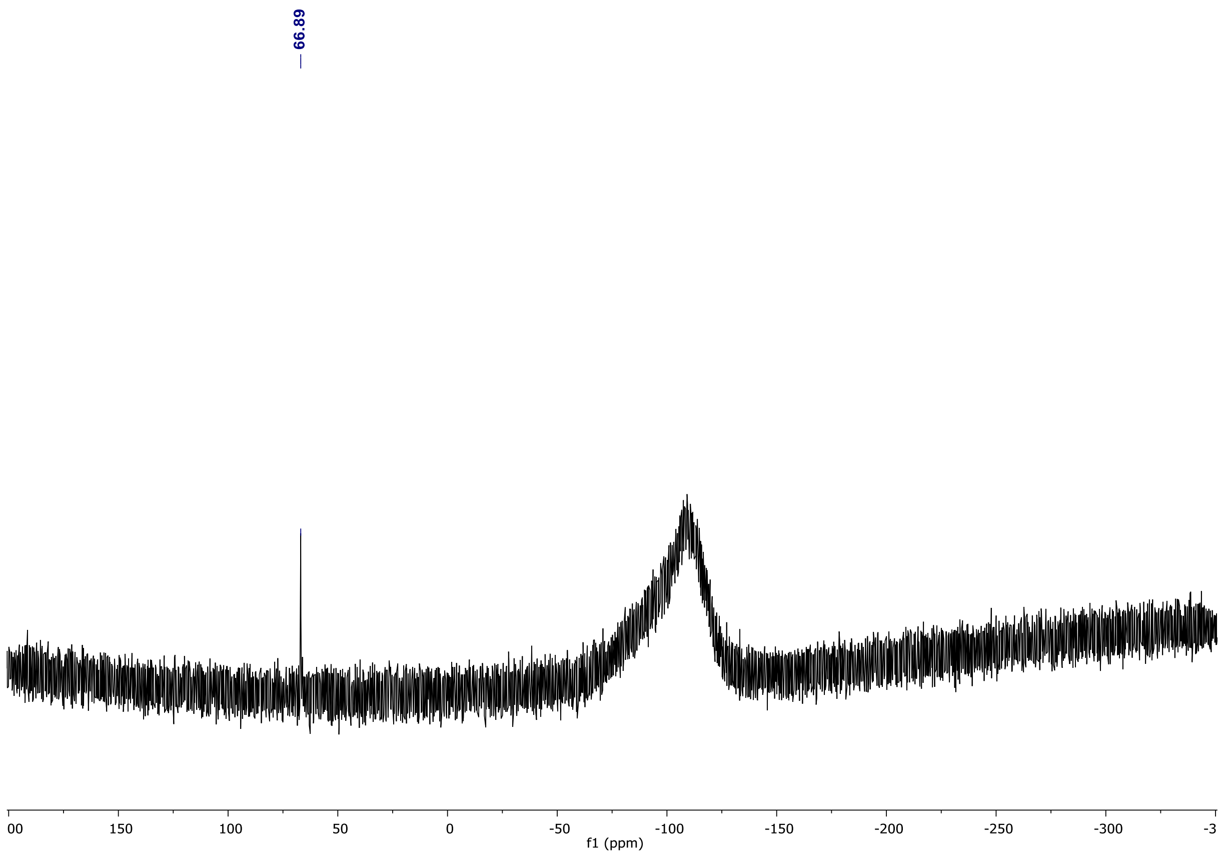

Figure S8. $^{29}Si\{^{1}H\}$ NMR spectrum of **1b** in DCM-d_2.

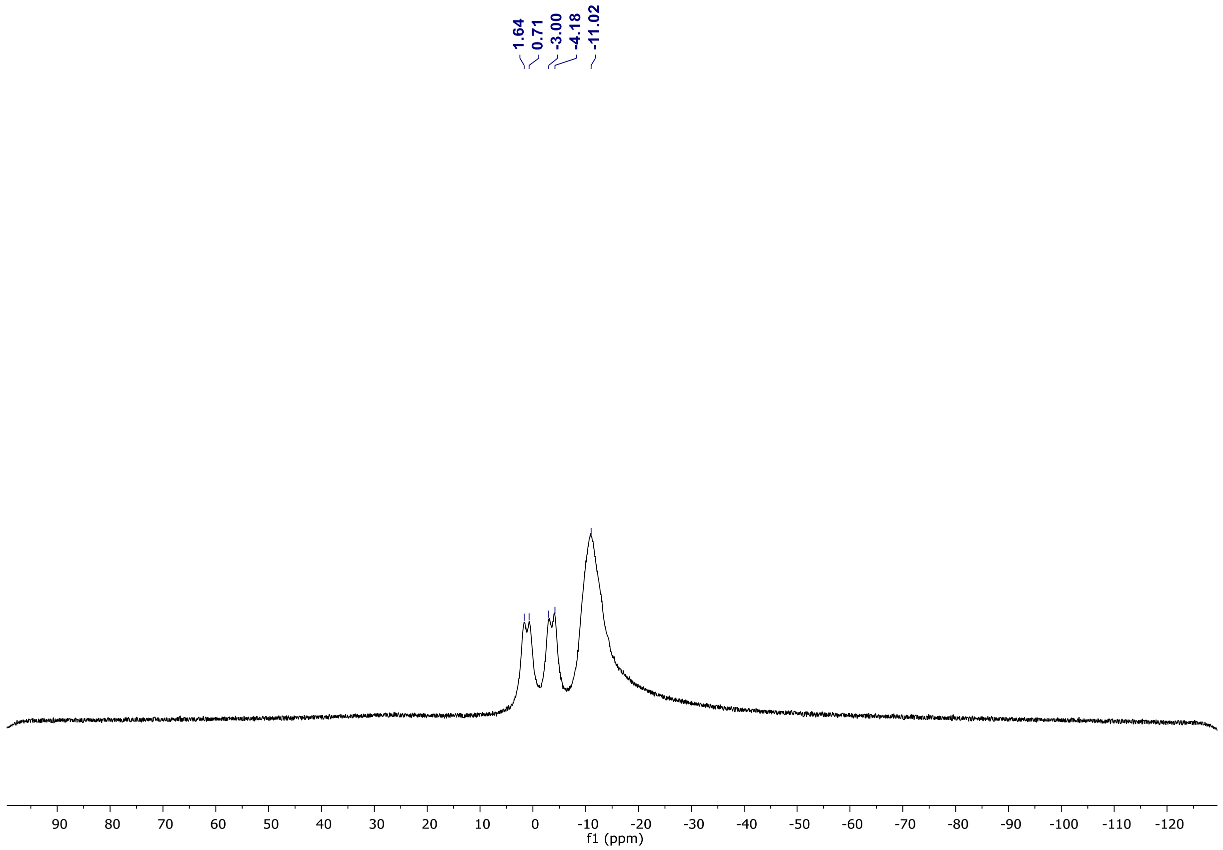

Figure S9. $^{11}B\{^{1}H\}$ NMR spectrum of **1b** in DCM-d_2.

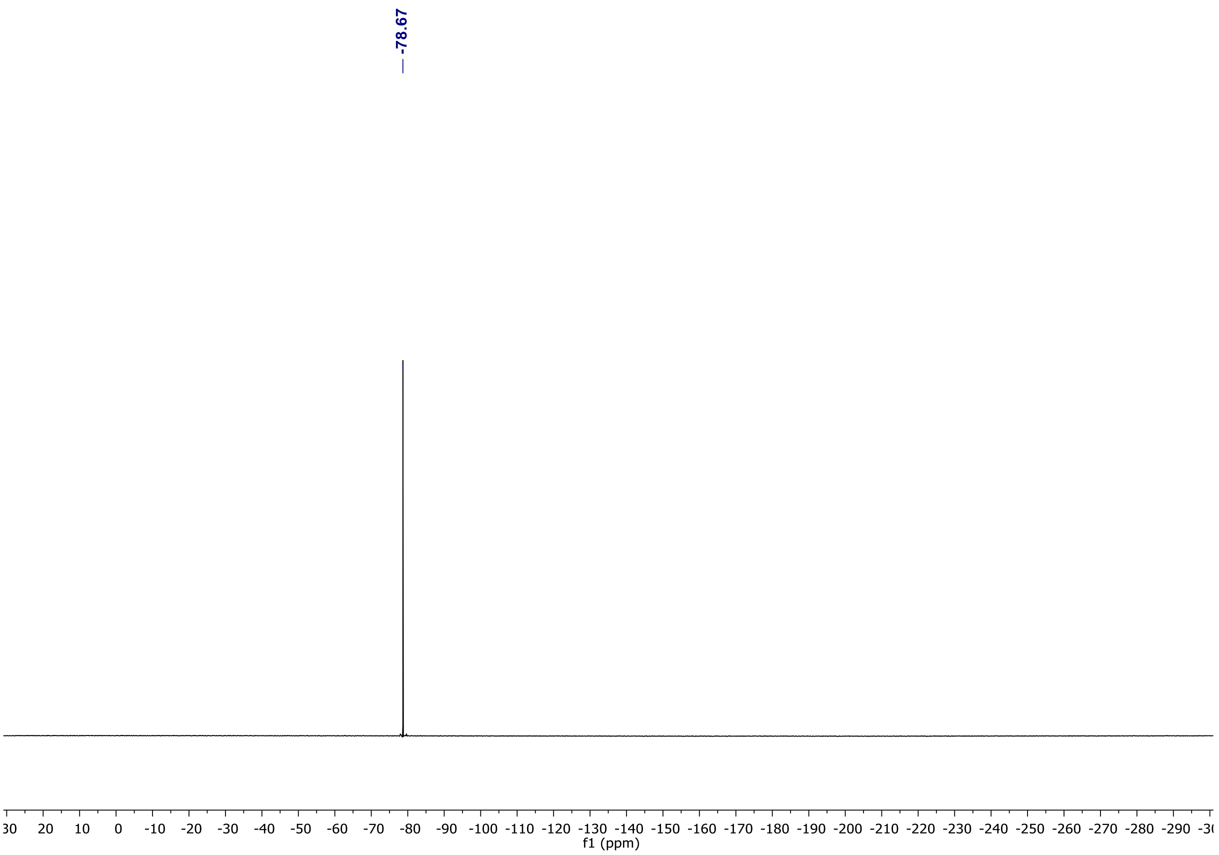

Figure S10. ^{19}F NMR spectrum of **1b** in DCM-d_2.

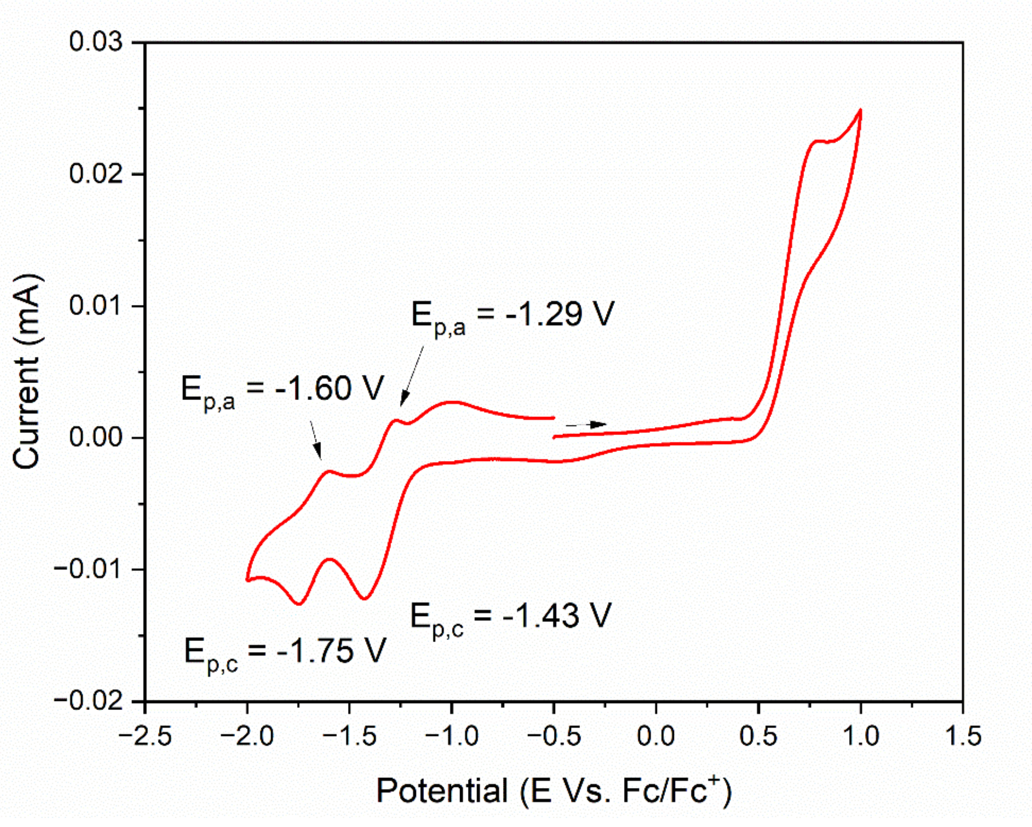

Figure S11. CV of complex **1b** at a scan rate of v = 100 mVs^{-1}. (1.5 mM in THF/ 0.1 M $TBAPF_6$). Initial potential E= -0.5 V vs. Fc/Fc^+.

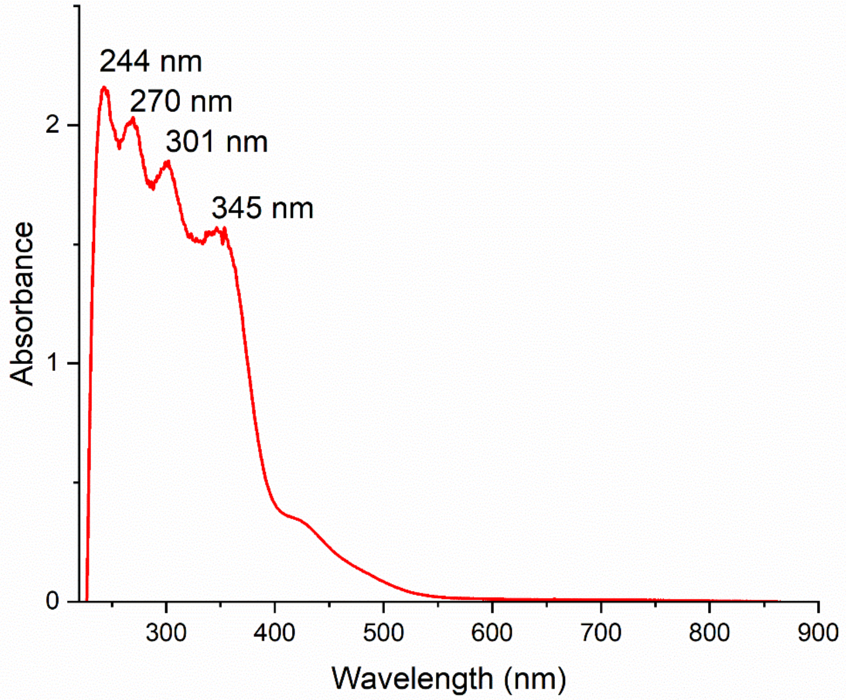

Figure S12. UV-Vis spectrum of **1b** in DCM.

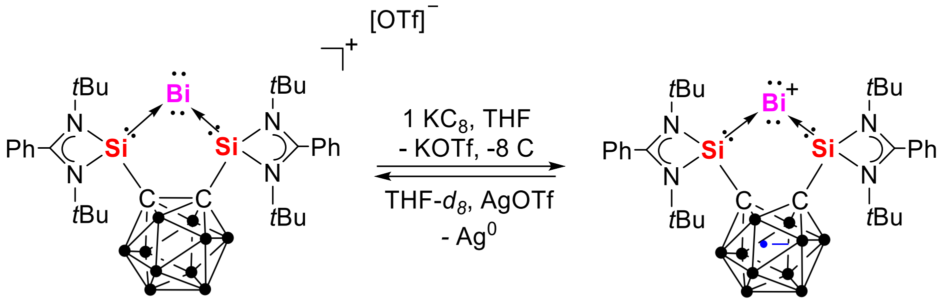

1b **2**

Synthesis of compound 2 (route A). To a Schlenk flask charged with **1b** (1020 mg, 1 mmol) and KC_8 (140 mg, 1.04 mmol) was added 60 mL THF at -30 °C with stirring. The reaction solution was allowed to warm to room temperature. After stirring for 2 h, the resulting suspension was filtered to give an orange solution and all volatiles were removed under vacuum and the residue was extracted with toluene (4 × 30 mL). Concentrating and cooling the solution at -30 °C afforded compound **2** as pale red crystals (375 mg, 43 % isolated yield). Single crystals of **2** suitable for X-ray diffraction analysis were obtained from a toluene solution at 4 °C. Compound **2** was treated with 1eq AgOTf in THF-d_8 at room temperature for 10 min, NMR analysis confirmed the quantitative regeneration of compound **1b**.

M.p. 200 °C (decomp.).

^{1}H NMR (400 MHz, THF-d_8) δ/ppm = 7.51 (br, Ar-H), 1.31 (br, C(CH_3)$_3$).

HR-MS (ESI): (m/z) calcd for $[M]^+$ ($C_{32}H_{56}B_{10}N_4Si_2Bi^+$): 870.4845; found: 870.4844.

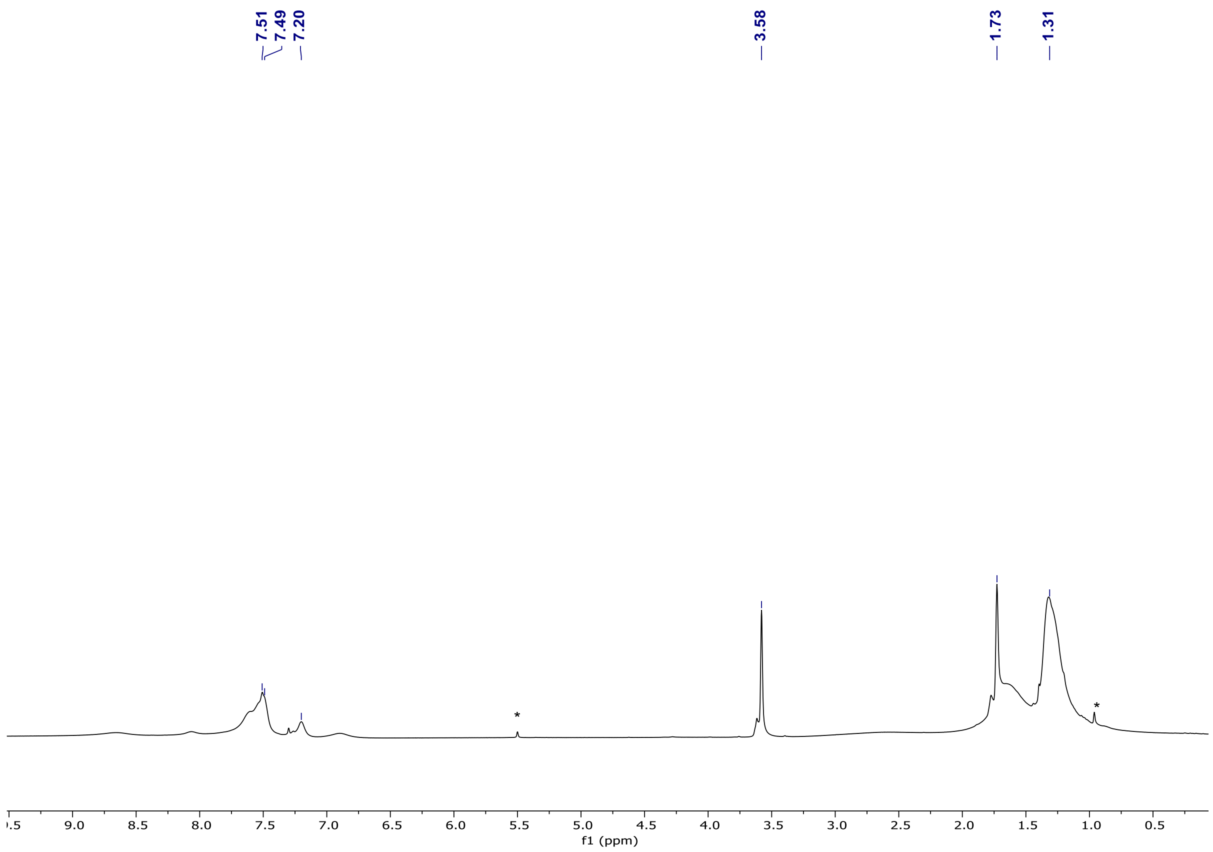

Figure S13. ^{1}H NMR spectrum of **2** in THF-d_8. *Unidentified impurities.

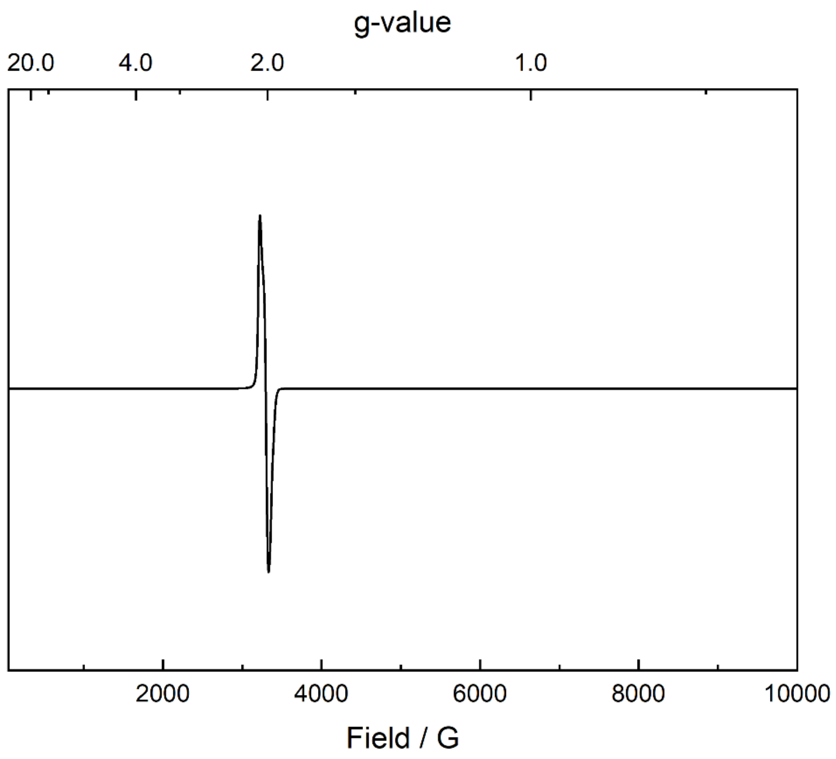

Figure S14. EPR spectrum of compound **2** in THF recorded at 10 K EPR. No additional signal compared to the spectrum at 293 K (Figure 4) was observed.

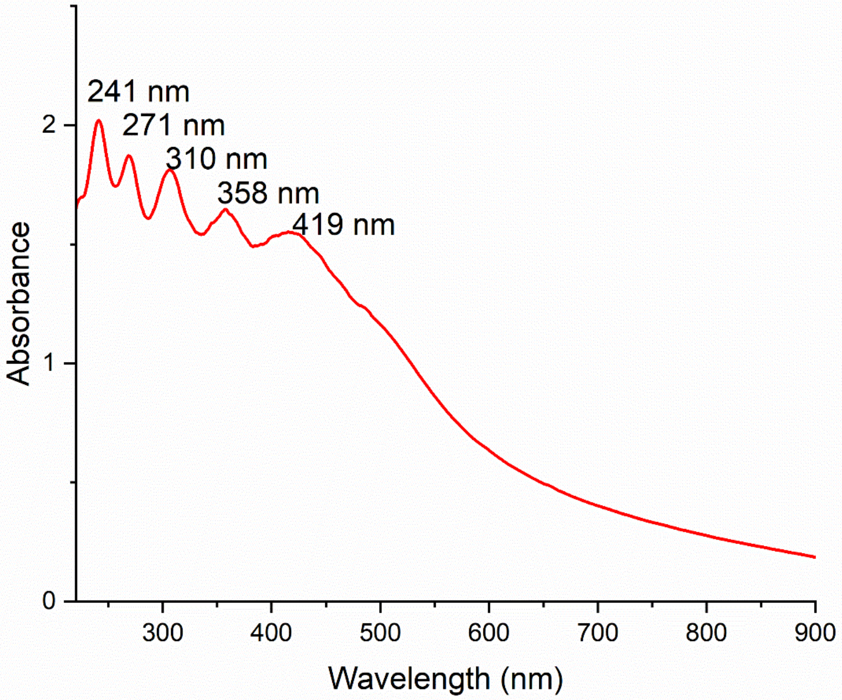

Figure S15. UV-Vis spectrum of **2** in THF.

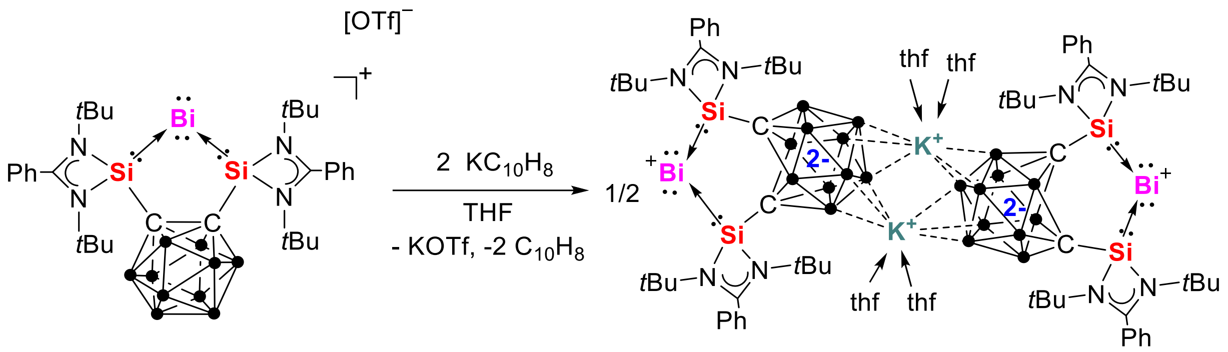

Synthesis of compound [3K(thf)$_2$]$_2$. To a cooled (-30 °C) solution of **1b** (1020 mg, 1 mmol) in 30 mL THF was added dropwise a potassium naphthalenide ($KC_{10}H_8$) THF solution prepared by stirring 77.3 mg potassium (2 mmol) and 254.5 mg naphthalene (2 mmol) in 15 mL THF at room temperature in over 2 h. The reaction solution was allowed to warm to room temperature. After stirring for 1 h, the resulting suspension was filtered to give an orange solution and all volatiles were then removed under vacuum and recrystallization from THF/Et_2O formed compound [**3**K(thf)$_2$]$_2$ (537 mg, 51 % isolated yield). In addition, compound [**3**K(thf)$_2$]$_2$ can be synthesized through the one-electron reduction of complex **2** with KC_8 in THF at room temperature. Single crystals of [**3**K(thf)$_2$]$_2$ suitable for X-ray diffraction analysis were obtained from a THF/Et_2O solution at room temperature.

M.p. 231.5 ^{o}C (decomp.).

^{1}H NMR (400 MHz, THF-d_8) δ/ppm = 7.56 (s, 2H, Ar-*H*), 7.47 (m, 8H, Ar-*H*), 1.29 (s, 36H, C(C*H*$_3$)$_3$). The 10 B*H* signals are very broad and unresolved.

^{13}C{^{1}H} NMR (101 MHz, THF-d_8) δ/ppm = 172.33 (s, N*C*N), 134.34, 131.26, 130.59, 130.43, 128.39, 126.31 (s, Ar-*C*), 55.15 (s, N*C*(CH$_3$)$_3$), 32.60 (s, NC(*C*H$_3$)$_3$). The carborane-*C* signal is very broad and unresolved.

^{29}Si{^{1}H} NMR (79 MHz, THF-d_8) δ/ppm = 51.80 (s).

^{11}B{^{1}H} NMR (160 MHz, DCM-d_2) δ/ppm = -26.39 – -6.03 (m, vb, *B*H).

Elemental analysis calcd for $C_{80}H_{144}B_{20}Bi_2K_2N_8O_4Si_4$: C, 45.61; H, 6.89; N, 5.32. Found: C, 41.07; H, 6.70; N, 5.50. [Consistently low C analysis may be due to the formation of silicon carbide].

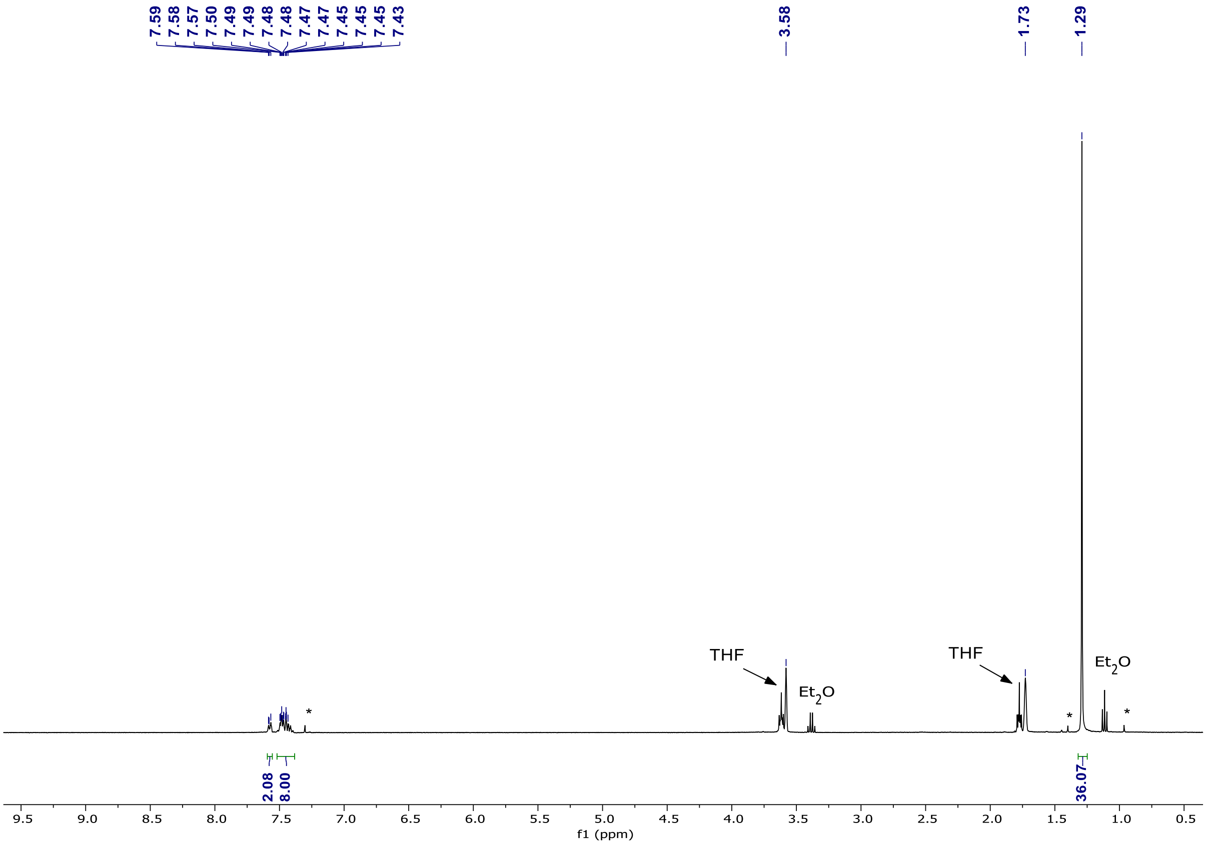

Figure S16. ^{1}H NMR spectrum of [**3**K(thf)$_2$]$_2$ in THF-*d*$_8$. *Unidentified impurities.

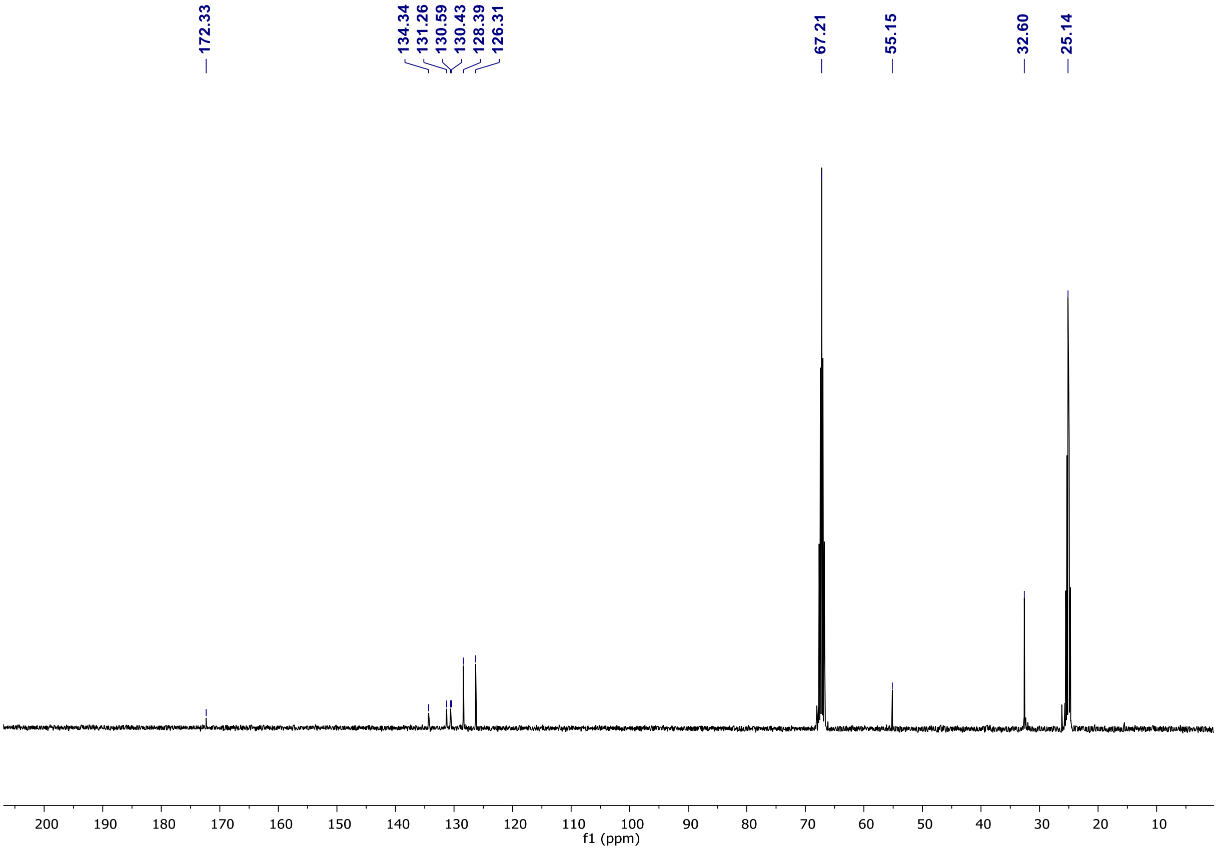

Figure S17. ^{13}C{^{1}H} NMR spectrum of [**3**K(thf)$_2$]$_2$ in THF-*d*$_8$.

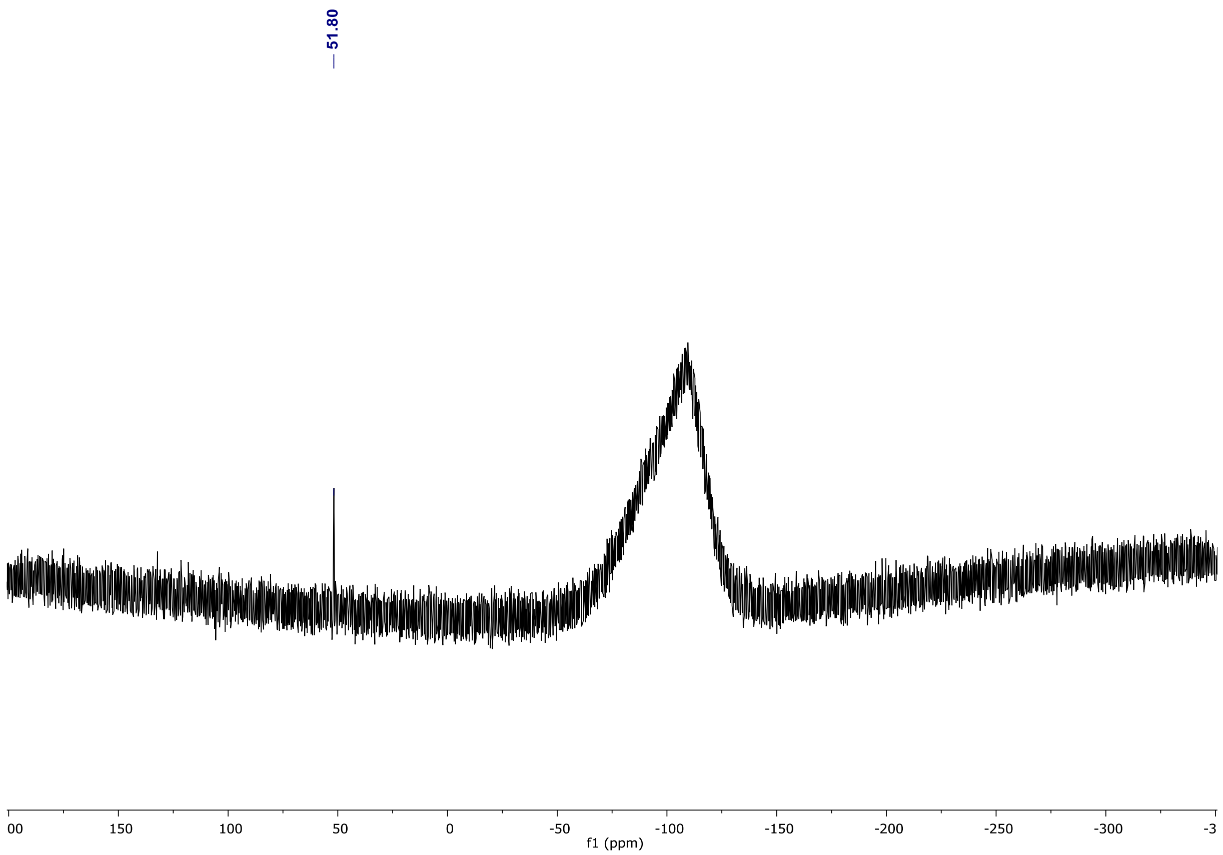

Figure S18. $^{29}Si\{^{1}H\}$ NMR spectrum of $[\mathbf{3}K(thf)_2]_2$ in THF-d_8.

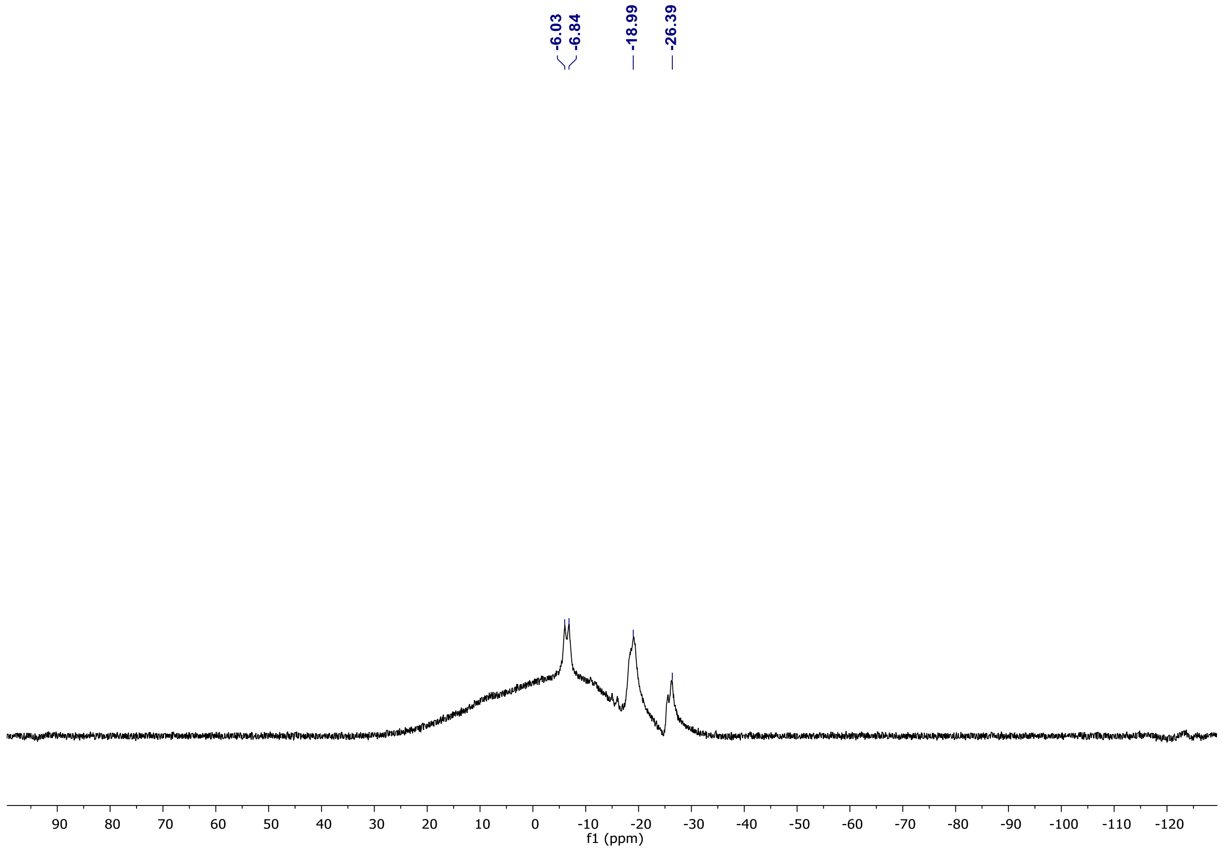

Figure S19. $^{11}B\{^{1}H\}$ NMR spectrum of $[\mathbf{3}K(thf)_2]_2$ in THF-d_8.

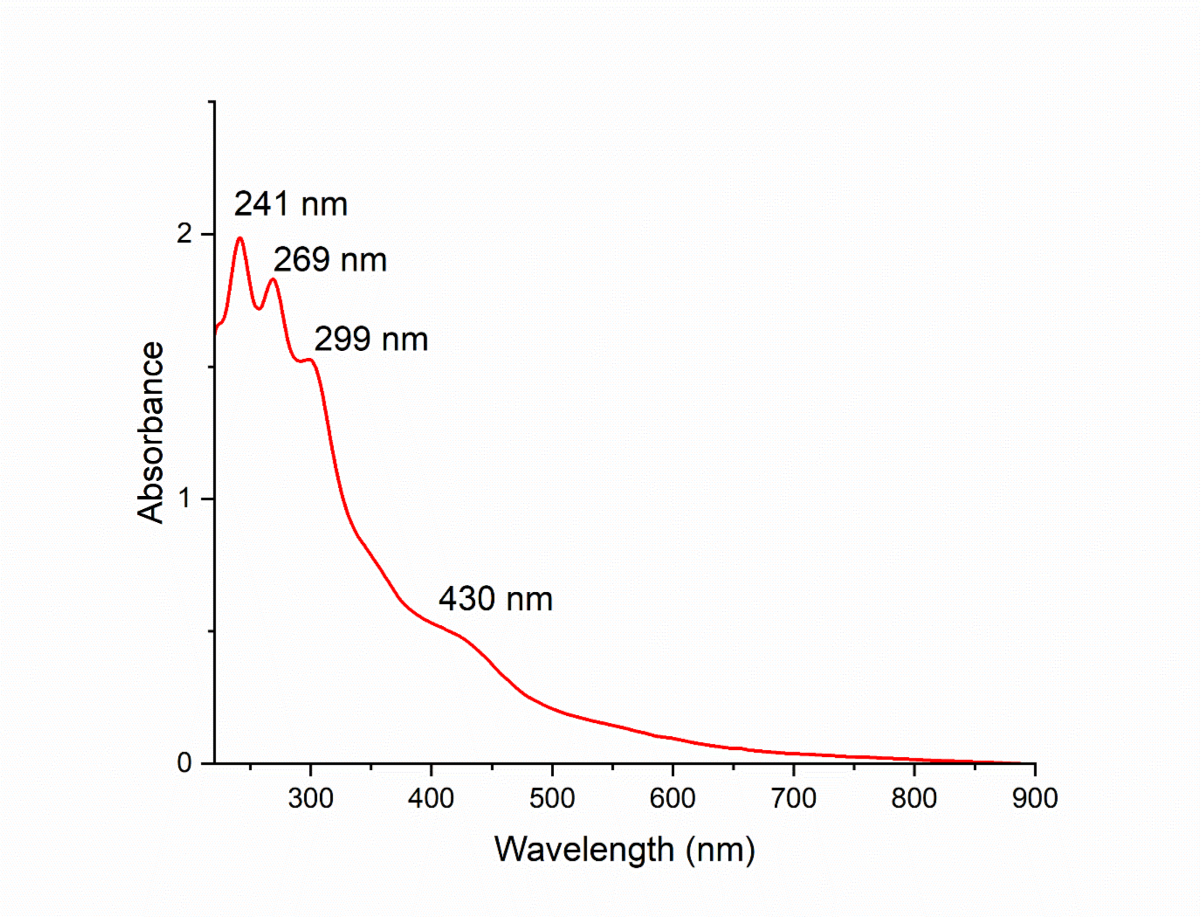

Figure S20. UV-Vis spectrum of [**3**K(thf)$_2$]$_2$ in THF.

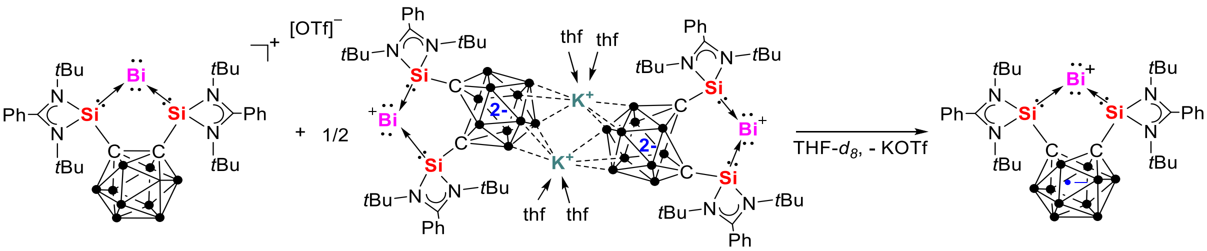

Synthesis of compound 2 (route B). Compound **2** could alternatively be obtained by treatment of compound [**3**K(thf)$_2$]$_2$ with **1b** as tested on an NMR scale.

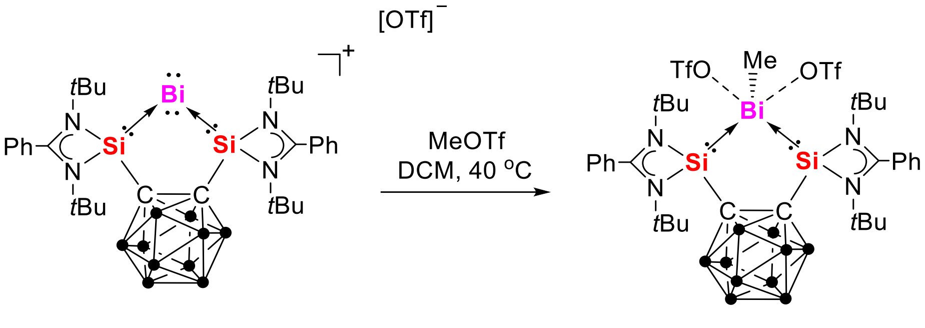

Synthesis of compound 4. To a mixture of compound **1b** (510 mg, 0.5 mmol) and 30 mL DCM in a 50 mL Schlenk flask was added 1.5 eq MeOTf (80 uL, 0.75 mmol) at room temperature under stirring. The color of the mixture changed to colorless over 4 h at 40 °C. Volatiles were then removed and recrystallization from DCM afforded compound **4** as colorless crystals (361 mg, 61% isolated yield). Colorless block crystals suitable for X-ray diffraction analysis were obtained from a concentrated DCM solution at room temperature.

M.p. 170 °C (decomp.).

^{1}H NMR (400 MHz, DCM-d_2) δ/ppm 8.16 (m, 2H, Ar-*H*), 7.66 (m, 4H, Ar-*H*), 7.57 (m, 2H, Ar-*H*), 7.41 – 7.36 (m, 2H, Ar-*H*), 2.47 (s, 3H, BiC*H*$_3$), 1.30 (d, *J* = 10.9 Hz, 36H, C(C*H*$_3$)$_3$). The 10 B*H* signals are very broad and unresolved.

^{13}C{^{1}H} NMR (101 MHz, DCM-d_2) δ/ppm = 184.88 (s, N*C*N), 132.80, 129.43, 129.18, 128.63, 128.60 (s, Ar-*C*), 120.74 (q, *J* = 320.1 Hz, *C*F$_3$), 85.68 (s, carborane-*C*), 58.83 (s, N*C*(CH$_3$)$_3$), 57.56 (s, N*C*(CH$_3$)$_3$), 32.29 (s, NC(*C*H$_3$)$_3$), 31.95 (s, NC(*C*H$_3$)$_3$), 8.12 (s, Bi*C*H$_3$).

^{29}Si{^{1}H} NMR (79 MHz, DCM-d_2) δ/ppm = 62.01 (s).

^{11}B{^{1}H} NMR (160 MHz, DCM-d_2) δ/ppm = -12.63 – 1.54 (m, vb).

^{19}F NMR (471 MHz, DCM-d_2) δ/ppm = -78.66 (s).

Elemental analysis calcd for $C_{35}H_{59}B_{10}Bi_1F_6N_4O_6S_2Si_2 \cdot CH_2Cl_2$: C, 34.09; H, 4.84; N, 4.41. Found: C, 34.23; H, 4.37; N, 3.96.

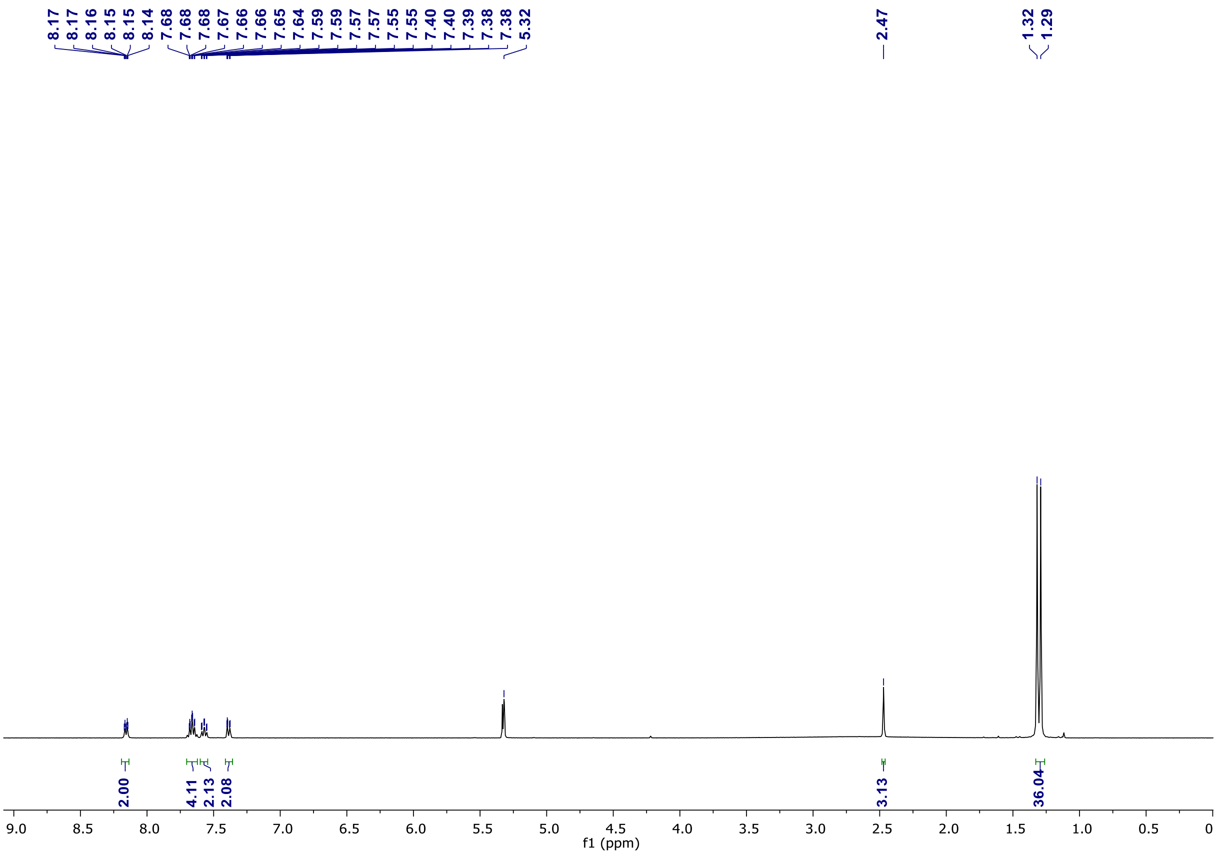

Figure S21. ^1H NMR spectrum of **4** in DCM-d_2.

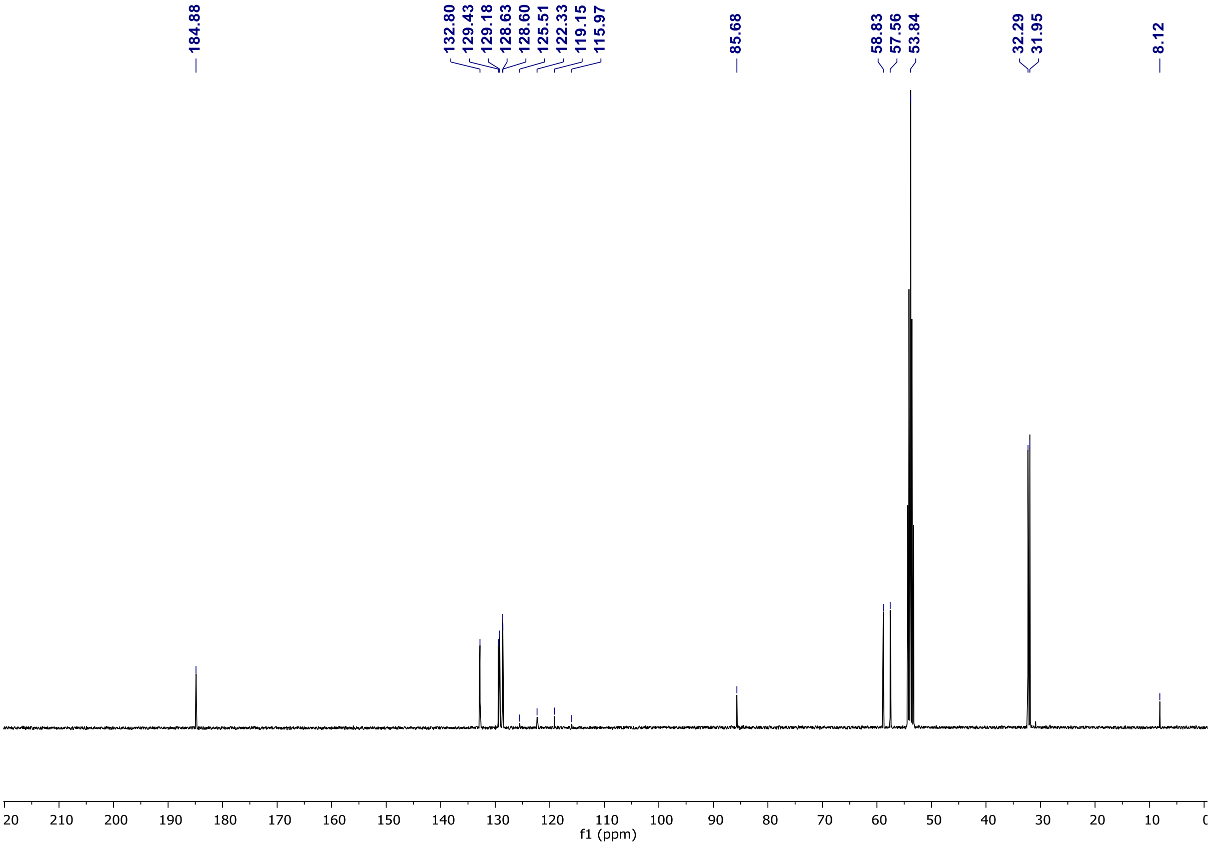

Figure S22. $^{13}C\{^1H\}$ NMR spectrum of **4** in DCM-d_2.

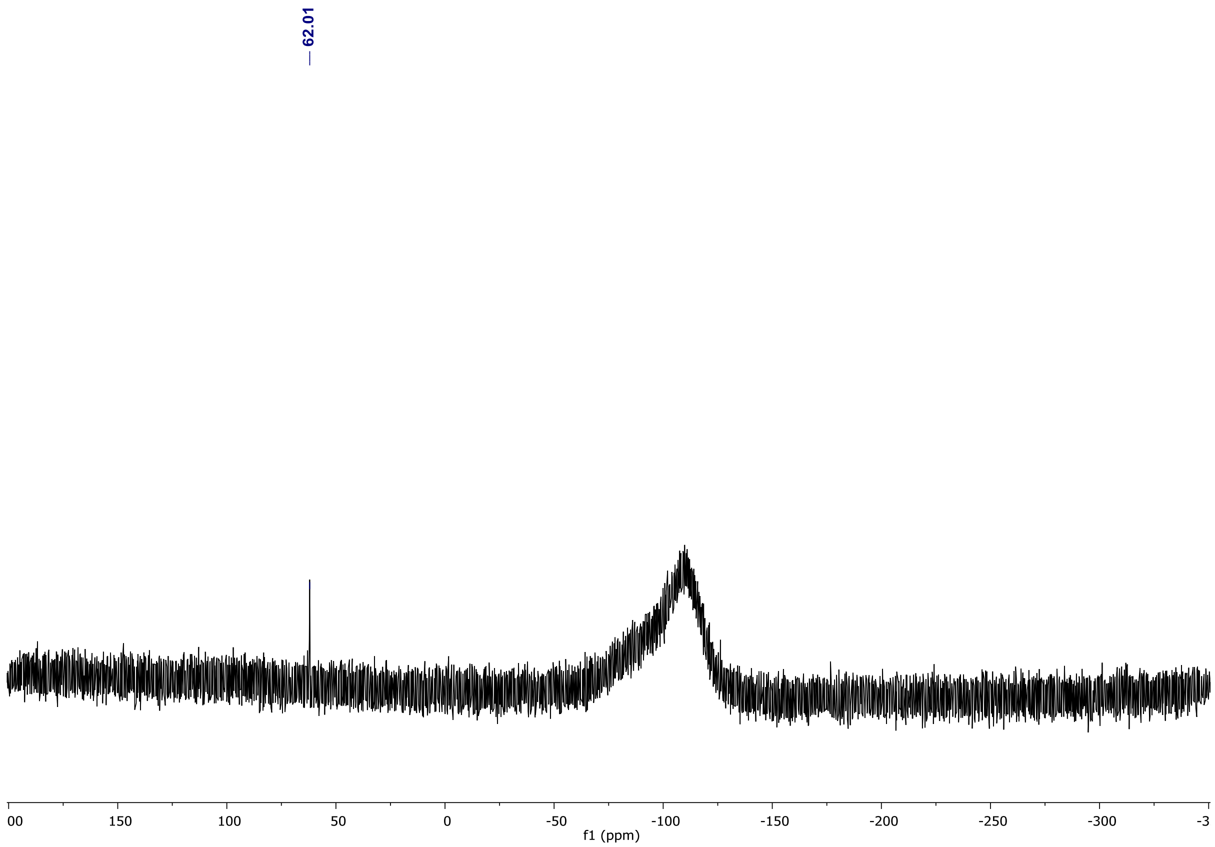

Figure S23. $^{29}Si\{^1H\}$ NMR spectrum of **4** in DCM-d_2.

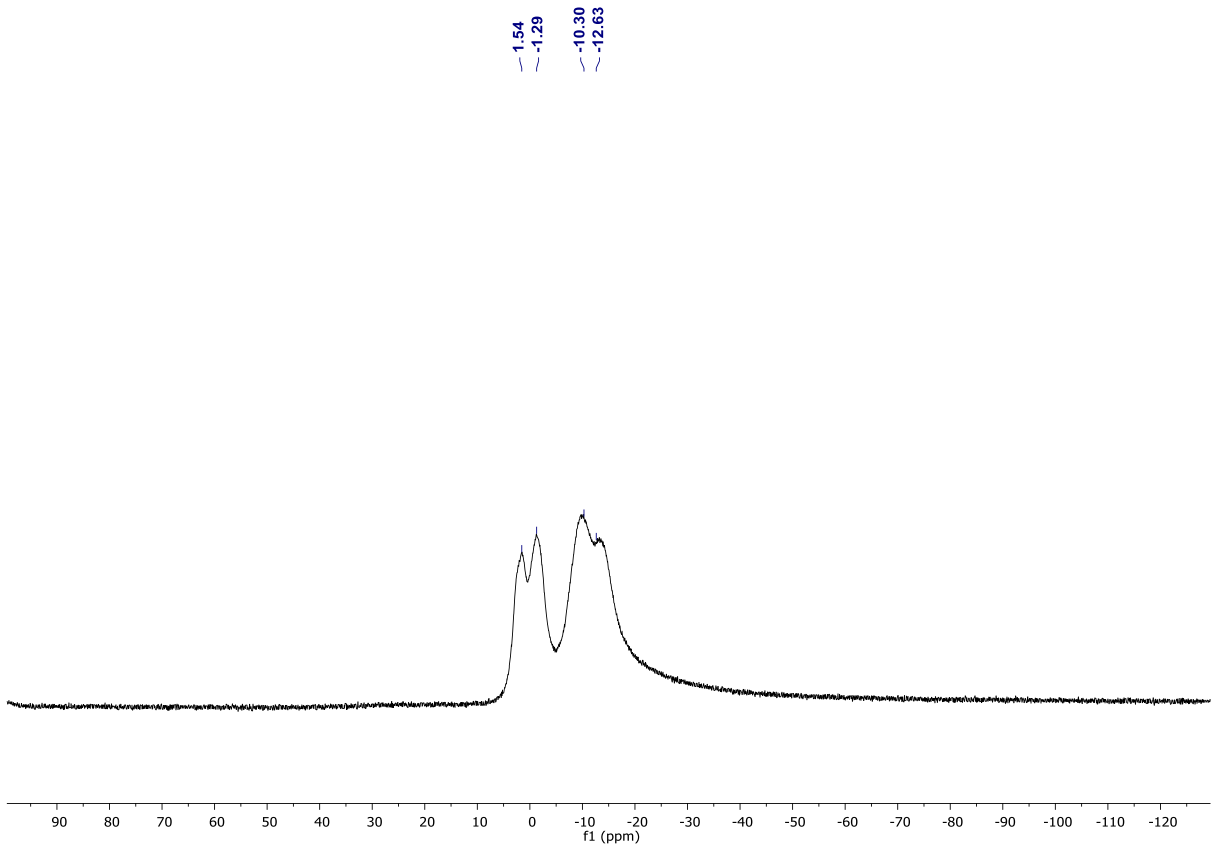

Figure S24. $^{11}B\{^1H\}$ NMR spectrum of **4** in DCM-d_2.

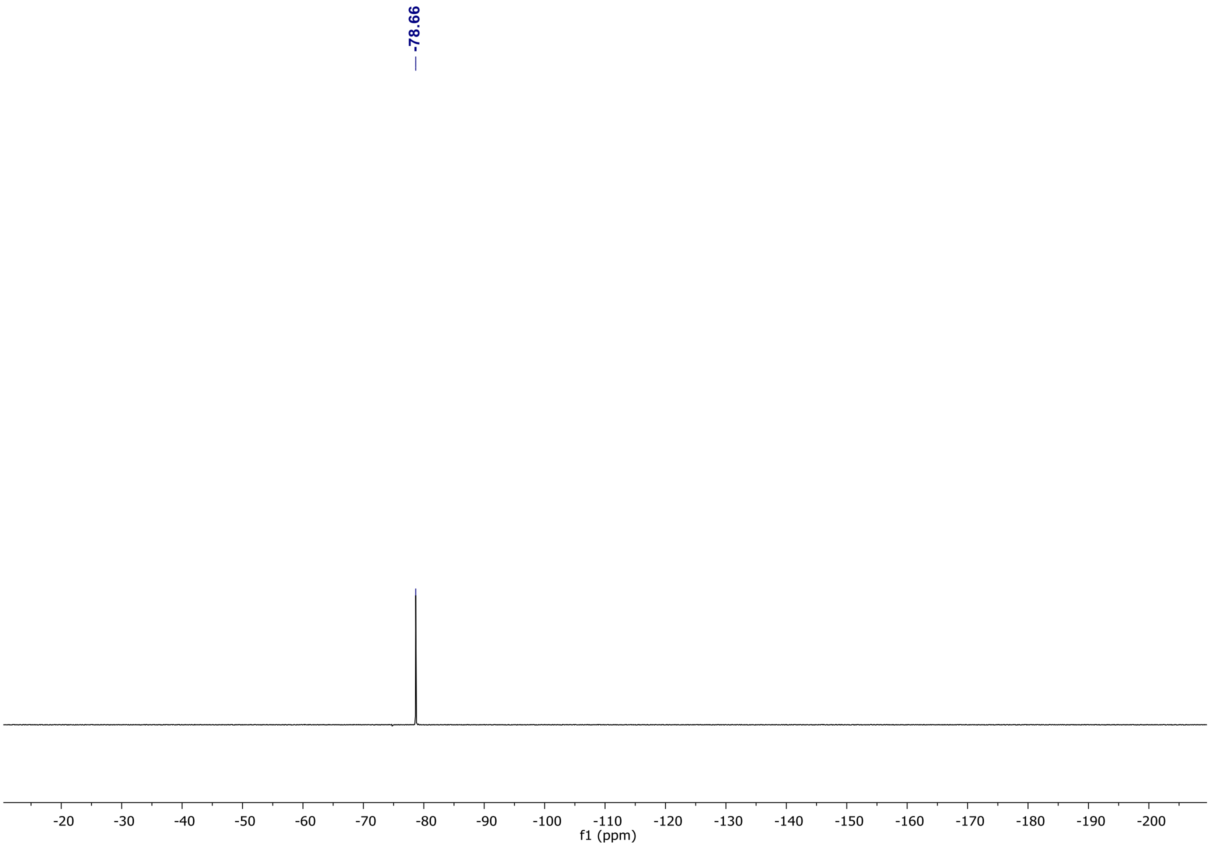

Figure S25. ^{19}F NMR spectrum of **4** in DCM-d_2.

C. X-ray Crystallographic Data

Table S1. Crystal data and structure refinement for **1a**.

Empirical formula	$C_{44} H_{68} B_{10} Bi I N_4 Si_2$	
Formula weight	1153.18	
Temperature	110.07(12) K	
Wavelength	1.54184 Å	
Crystal system	Monoclinic	
Space group	$P\ 2_1/c$	
Unit cell dimensions	a = 13.6332(2) Å	α = 90°.
	b = 18.0507(3) Å	β = 95.2830(10)°.
	c = 21.0915(3) Å	γ = 90°.
Volume	5168.33(14) $Å^3$	
Z	4	
Density (calculated)	1.482 Mg/m^3	
Absorption coefficient	12.077 mm^{-1}	
F(000)	2296	
Crystal size	0.04 x 0.03 x 0.02 mm^3	
Theta range for data collection	3.228 to 72.510°.	
Index ranges	-14<=h<=16, -22<=k<=20, -25<=l<=26	
Reflections collected	20747	
Independent reflections	9991 [R(int) = 0.0216]	
Completeness to theta = 67.684°	99.9 %	
Absorption correction	Semi-empirical from equivalents	
Max. and min. transmission	1.00000 and 0.42040	
Refinement method	Full-matrix least-squares on F^2	
Data / restraints / parameters	9991 / 0 / 463	
Goodness-of-fit on F^2	1.027	
Final R indices [I>2sigma(I)]	R1 = 0.0319, wR2 = 0.0800	
R indices (all data)	R1 = 0.0345, wR2 = 0.0818	
Extinction coefficient	n/a	
Largest diff. peak and hole	2.126 and -1.769 e.$Å^{-3}$	

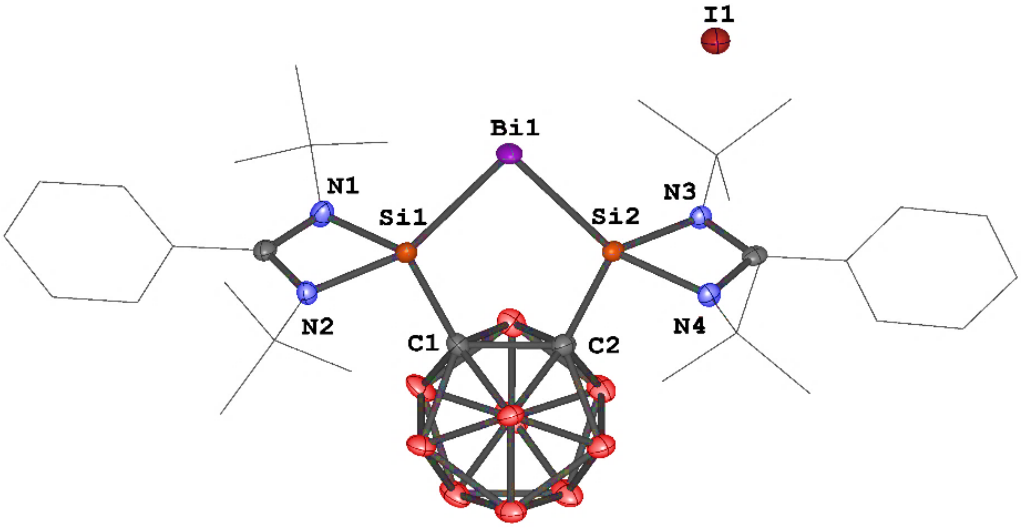

Figure S26. Molecular structure of **1a**. Thermal ellipsoids are drawn at the 50% probability level. H atoms are omitted for clarity.

Table S2. Selected interatomic distances and angles of compound **1a.**

Bond lengths [Å]		Angles [°]	
Bi(1)-Si(2)	2.5958(9)	Si(1)-Bi(1)-Si(2)	79.28(3)
Bi(1)-Si(1)	2.5940(10)	C(1)-Si(1)-Bi(1)	115.14(11)
C(2)-C(1)	1.691(5)	C(2)-Si(2)-Bi(1)	115.65(11)
Si(2)-C(2)	1.937(4)		
Si(1)-C(1)	1.937(4)		

Table S3. Crystal data and structure refinement for **1b**.

Empirical formula	$C_{33} H_{56} B_{10} Bi F_3 N_4 O_3 S Si_2$	
Formula weight	1019.13	
Temperature	110.15 K	
Wavelength	1.54184 Å	
Crystal system	Monoclinic	
Space group	$P\,2_1/n$	
Unit cell dimensions	a = 15.66060(10) Å	α = 90°.
	b = 10.19510(10) Å	β = 93.6960(10)°.
	c = 27.7238(2) Å	γ = 90°.
Volume	4417.21(6) $Å^3$	
Z	4	
Density (calculated)	1.532 Mg/m^3	
Absorption coefficient	9.212 mm^{-1}	
F(000)	2040	
Crystal size	0.06 x 0.03 x 0.02 mm^3	
Theta range for data collection	3.157 to 72.661°.	
Index ranges	-19<=h<=14, -12<=k<=12, -34<=l<=33	
Reflections collected	31319	
Independent reflections	8661 [R(int) = 0.0289]	
Completeness to theta = 67.684°	100.0 %	
Absorption correction	Semi-empirical from equivalents	
Max. and min. transmission	1.00000 and 0.24536	
Refinement method	Full-matrix least-squares on F^2	
Data / restraints / parameters	8661 / 0 / 526	
Goodness-of-fit on F^2	1.040	
Final R indices [I>2sigma(I)]	R1 = 0.0228, wR2 = 0.0591	
R indices (all data)	R1 = 0.0240, wR2 = 0.0600	
Extinction coefficient	n/a	
Largest diff. peak and hole	1.129 and -1.081 e.$Å^{-3}$	

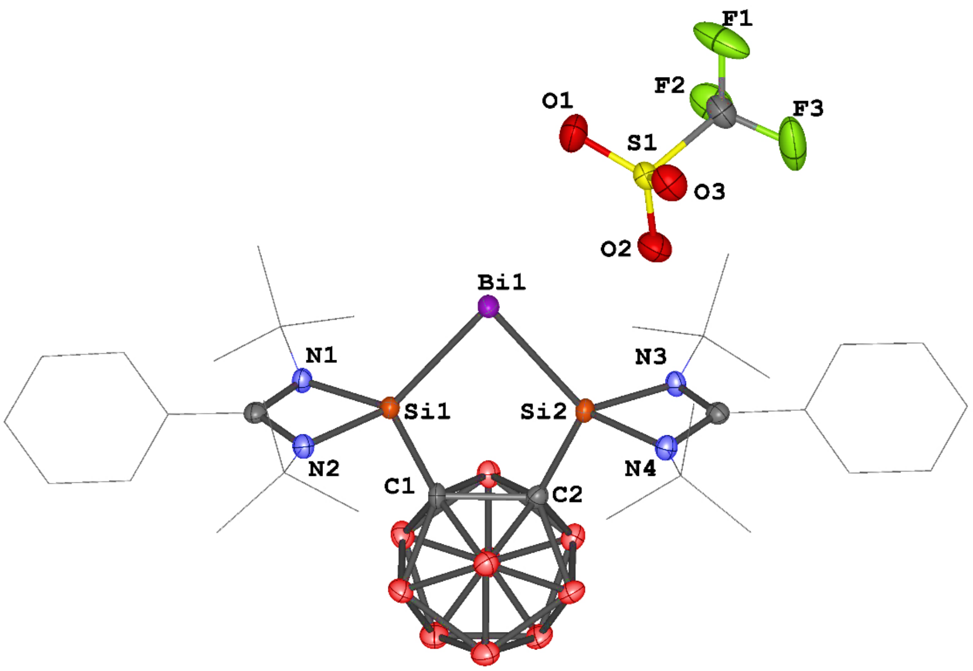

Figure S27. Molecular structure of compound **1b**. Thermal ellipsoids are drawn at the 50% probability level. H atoms are omitted for clarity.

Table S4. Selected interatomic distances and angles of compound **1b.**

Bond lengths [Å]		Angles [°]	
Bi(1)-Si(2)	2.5774(6)	Si(1)-Bi(1)-Si(2)	79.486(18)
Bi(1)-Si(1)	2.5931(6)	C(1)-Si(1)-Bi(1)	115.25(7)
C(2)-C(1)	1.692(3)	C(2)-Si(2)-Bi(1)	115.53(7)
Si(1)-C(1)	1.923(2)		
Si(2)-C(2)	1.925(2)		

Table S5. Crystal data and structure refinement for **2**.

Empirical formula	$C_{46} H_{72} B_{10} Bi N_4 Si_2$	
Formula weight	1054.33	
Temperature	111.15 K	
Wavelength	1.54184 Å	
Crystal system	Orthorhombic	
Space group	*Cmcm*	
Unit cell dimensions	a = 23.2329(4) Å	α = 90°.
	b = 18.6010(4) Å	β = 90°.
	c = 13.6308(3) Å	γ = 90°.
Volume	5890.6(2) $Å^3$	
Z	4	
Density (calculated)	1.189 Mg/m^3	
Absorption coefficient	6.487 mm^{-1}	
F(000)	2148	
Crystal size	0.08 x 0.04 x 0.02 mm^3	
Theta range for data collection	3.805 to 72.824°.	
Index ranges	-20<=h<=28, -20<=k<=22, -16<=l<=16	
Reflections collected	21230	
Independent reflections	3118 [R(int) = 0.0935]	
Completeness to theta = 67.684°	99.8 %	
Absorption correction	Semi-empirical from equivalents	
Max. and min. transmission	1.00000 and 0.46601	
Refinement method	Full-matrix least-squares on F^2	
Data / restraints / parameters	3118 / 0 / 136	
Goodness-of-fit on F^2	1.096	
Final R indices [I>2sigma(I)]	R1 = 0.0561, wR2 = 0.1501	
R indices (all data)	R1 = 0.0611, wR2 = 0.1545	
Extinction coefficient	n/a	
Largest diff. peak and hole	2.404 and -1.571 e.$Å^{-3}$	

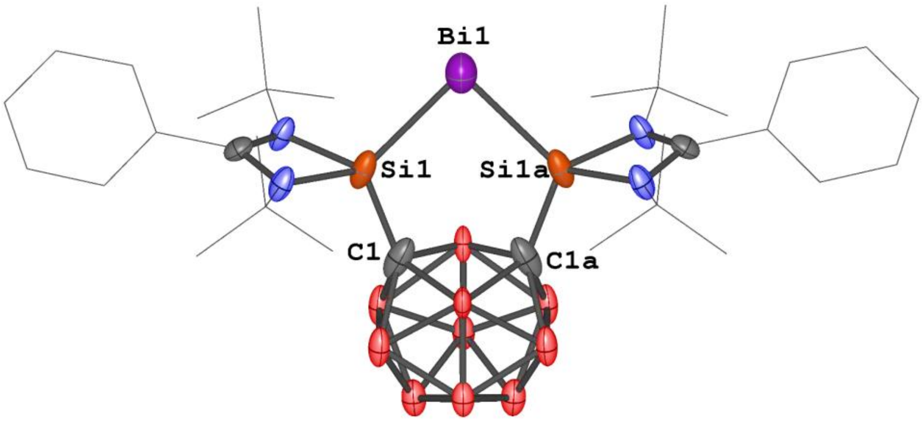

Figure S28. Molecular structure of compound **2**. Thermal ellipsoids are drawn at the 50% probability level. H atoms are omitted for clarity.

Table S6. Selected interatomic distances and angles of compound **2.**

Bond lengths [Å]		Angles [°]	
C(1)-Si(1)	1.851(8)	Si(1)-Bi(1)-Si(1a)	86.25(8)
Si(1)-Bi(1)	2.576(2)	C(1)-Si(1)-Bi(1)	117.1(3)
C(1).......C(1a)	2.268		

Table S7. Crystal data and structure refinement for [**3**K(thf)$_2$]$_2$.

Empirical formula	C_{88} H_{160} B_{20} Bi_2 K_2 N_8 O_6 Si_4	
Formula weight	2250.95	
Temperature	111.5(6) K	
Wavelength	1.54184 Å	
Crystal system	Triclinic	
Space group	*P*-1	
Unit cell dimensions	a = 10.0567(4) Å	α = 70.800(4)°.
	b = 14.8404(6) Å	β = 86.199(3)°.
	c = 19.9981(8) Å	γ = 84.365(3)°.
Volume	2803.2(2) Å^3	
Z	1	
Density (calculated)	1.333 Mg/m^3	
Absorption coefficient	7.539 mm^{-1}	
F(000)	1152	
Crystal size	0.06 x 0.03 x 0.02 mm^3	
Theta range for data collection	3.164 to 72.691°.	
Index ranges	-9<=h<=12, -18<=k<=16, -24<=l<=24	
Reflections collected	21148	
Independent reflections	10794 [R(int) = 0.0667]	
Completeness to theta = 67.684°	99.8 %	
Absorption correction	Semi-empirical from equivalents	
Max. and min. transmission	1.00000 and 0.85827	
Refinement method	Full-matrix least-squares on F^2	
Data / restraints / parameters	10794 / 73 / 642	
Goodness-of-fit on F^2	1.034	
Final R indices [I>2sigma(I)]	R1 = 0.0448, wR2 = 0.1134	
R indices (all data)	R1 = 0.0526, wR2 = 0.1207	
Extinction coefficient	n/a	
Largest diff. peak and hole	1.752 and -2.348 e.Å^{-3}	

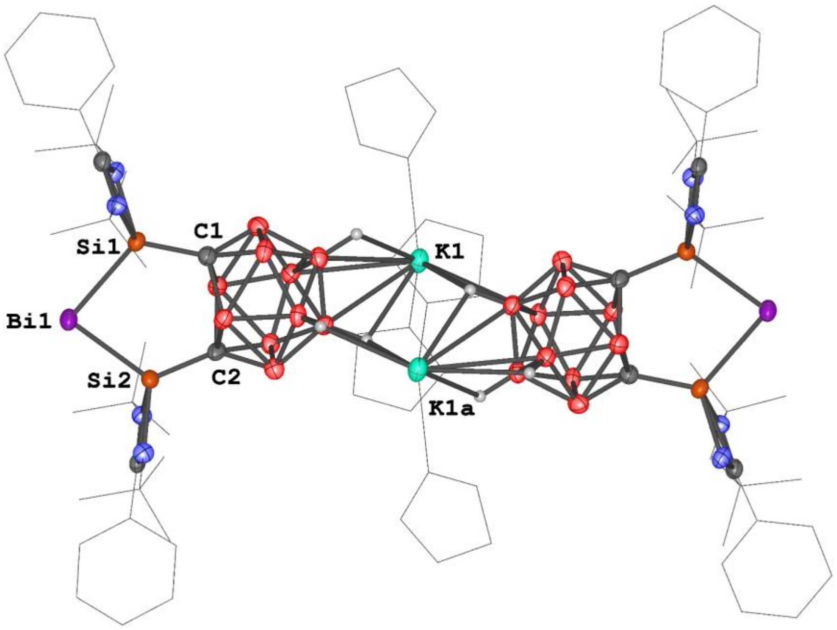

Figure S29. Molecular structure of [**3**K(thf)$_2$]$_2$. Thermal ellipsoids are drawn at the 50% probability level. H atoms and free solvent THF are omitted for clarity.

Table S8. Selected interatomic distances and angles of compound [**3**K(thf)$_2$]$_2$.

Bond lengths [Å]		Angles [°]	
Bi(1)-Si(1)	2.6266(13)	Si(2)-Bi(1)-Si(1)	90.27(4)
Bi(1)-Si(2)	2.6138(13)	C(1)-Si(1)-Bi(1)	116.52(16)
K(1)-B(10)	3.321(6)	C(2)-Si(2)-Bi(1)	115.93(16)
K(1)-B(8)	3.210(6)		
K(1)-B(9)	3.223(6)		
C(1) ....C(2)	2.576		
Si(1)-C(1)	1.786(5)		
Si(2)-C(2)	1.789(5)		

Table S9. Crystal data and structure refinement for **4**.

Empirical formula	$C_{36}H_{61}B_{10}BiCl_2F_6N_4O_6S_2Si_2$	
Formula weight	1268.16	
Temperature	111.15 K	
Wavelength	1.54184 Å	
Crystal system	Monoclinic	
Space group	P 1 2_1/n 1	
Unit cell dimensions	a = 16.1469(4) Å	α = 90°.
	b = 18.0810(3) Å	β = 98.144(2)°.
	c = 18.3593(4) Å	γ = 90°.
Volume	5306.0(2) $Å^3$	
Z	4	
Density (calculated)	1.588 Mg/m^3	
Absorption coefficient	9.186 mm^{-1}	
F(000)	2536	
Crystal size	0.05 x 0.04 x 0.02 mm^3	
Theta range for data collection	3.414 to 72.874°.	
Index ranges	-19<=h<=19, -22<=k<=15, -22<=l<=18	
Reflections collected	36892	
Independent reflections	10398 [R(int) = 0.0906]	
Completeness to theta = 67.684°	100.0 %	
Absorption correction	Semi-empirical from equivalents	
Max. and min. transmission	1.00000 and 0.61545	
Refinement method	Full-matrix least-squares on F^2	
Data / restraints / parameters	10398 / 6 / 639	
Goodness-of-fit on F^2	1.042	
Final R indices [I>2sigma(I)]	R1 = 0.0620, wR2 = 0.1785	
R indices (all data)	R1 = 0.0799, wR2 = 0.2150	
Extinction coefficient	n/a	
Largest diff. peak and hole	3.143 and -2.912 e.$Å^{-3}$	

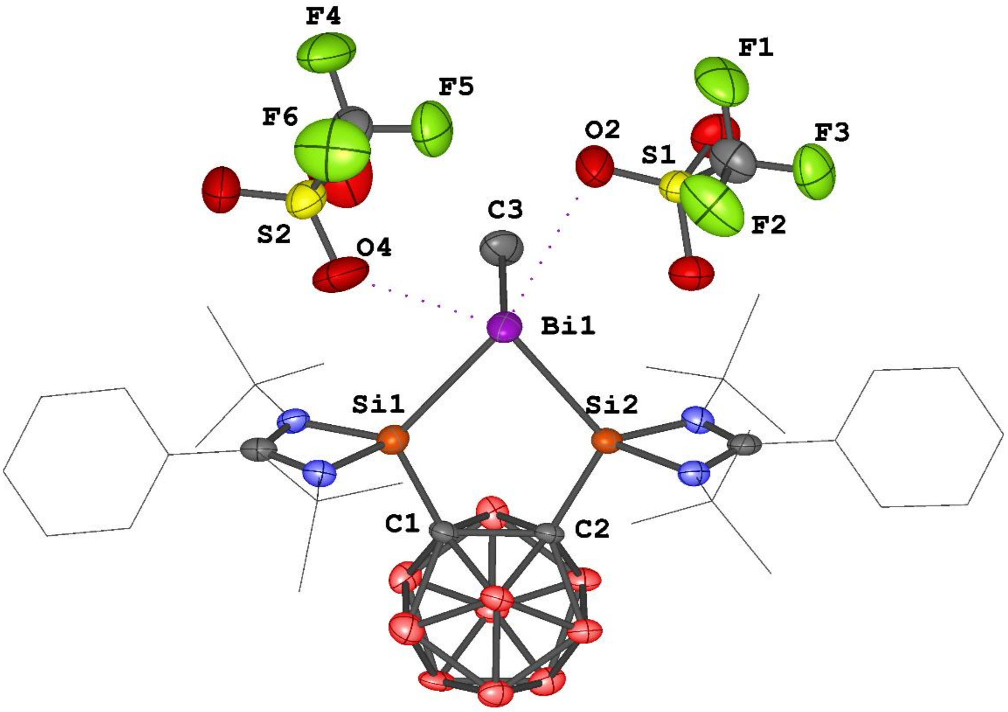

Figure S30. Molecular structure of **4**. Thermal ellipsoids are drawn at the 50% probability level. H atoms and solvent DCM are omitted for clarity.

Table S10. Selected interatomic distances and angles of compound **4.**

Bond lengths [Å]		Angles [°]	
Bi(1)-Si(2)	2.7003(17)	Si(1)-Bi(1)-Si(2)	80.44(5)
Bi(1)-Si(1)	2.7160(19)	C(1)-Si(1)-Bi(1)	112.1(2)
Bi(1)-C(3)	2.278(9)	C(2)-Si(2)-Bi(1)	112.24(19)
C(2)-C(1)	1.717(9)	C(3)-Bi(1)-Si(1)	96.5(2)
Si(1)-C(1)	1.929(7)	C(3)-Bi(1)-Si(2)	94.7(3)
Si(2)-C(2)	1.917(6)		

D. Theoretical Calculations

D1. Computational Details

Single-point calculations on the XRD structures of compounds **1a**, **2**, and **3** were done using the B3LYP[5] and CAM-B3LYP[6] functionals in combination with the def2-TZVPD[7] basis set on Gaussian 16.[8] This basis set uses an effective core potential to describe the inner 60 electrons of Bismuth.[9] Additionally, the geometry of compound **2** was further optimized at the B3LYP-D3BJ[10] and CAM-B3LYP-D3BJ[5] / def2-TZVPD levels of theory. Frequency calculations confirmed the achieved geometries are stable minima, as no negative values were obtained for their second derivatives. We did electronic structure analyses on all the geometries/levels of theory employing the quantum theory of atoms in molecules (QTAIM) partition to compute atomic charges and localization/delocalization indices using the AIMAll (Version 19.10.12) package.[11]

D2. Analyses on XRD structures

CAM-B3LYP functional

**QTAIM charges/ LI(C_n) & DIs**	**1a**	**2**	**3**
Bi	0.10	-0.31	-0.45
C_1	-1.75	-2.28	-2.53
C_2	-1.74	-2.28	-2.54
B-H cage	1.98	2.28	1.99
Si_1	1.81	2.06	2.11
Si_2	1.93	2.06	2.09
Silylenyl ligand	1.06	1.29	1.27
C1	5.59	6.19	6.47
C2	5.59	6.19	6.47
B_1-C_1	0.42	0.46	0.47
C_1-C_2	0.97	0.38	0.22
B_2-C_1	0.52	0.56	0.55
Si_1-C_1	0.49	0.56	0.63
Bi-Si_1	1.01	1.01	0.94
B_1'-C_2	0.41	0.46	0.47
B_3-C_2	0.51	0.56	0.55
Si_2-C_2	0.48	0.56	0.63
Bi-Si_2	1.03	1.01	0.96

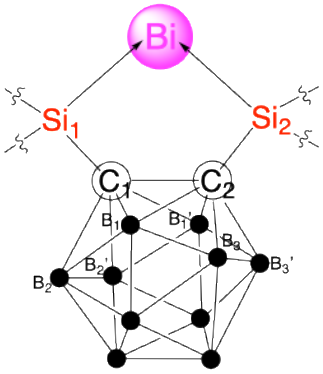

Figure S31: QTAIM charges (in blue) and Localization and Delocalization Indices (LIs & DIs) (in black) of selected atoms of XRD Bismuth complexes **1a**, **2**, and **3** obtained at the **CAM-B3LYP**/def2-TZVPD level of theory. Shaded cells correspond to bonded atom pairs according to the QTAIM analysis. Atom labels follow the classification depicted in the sketch on the right.

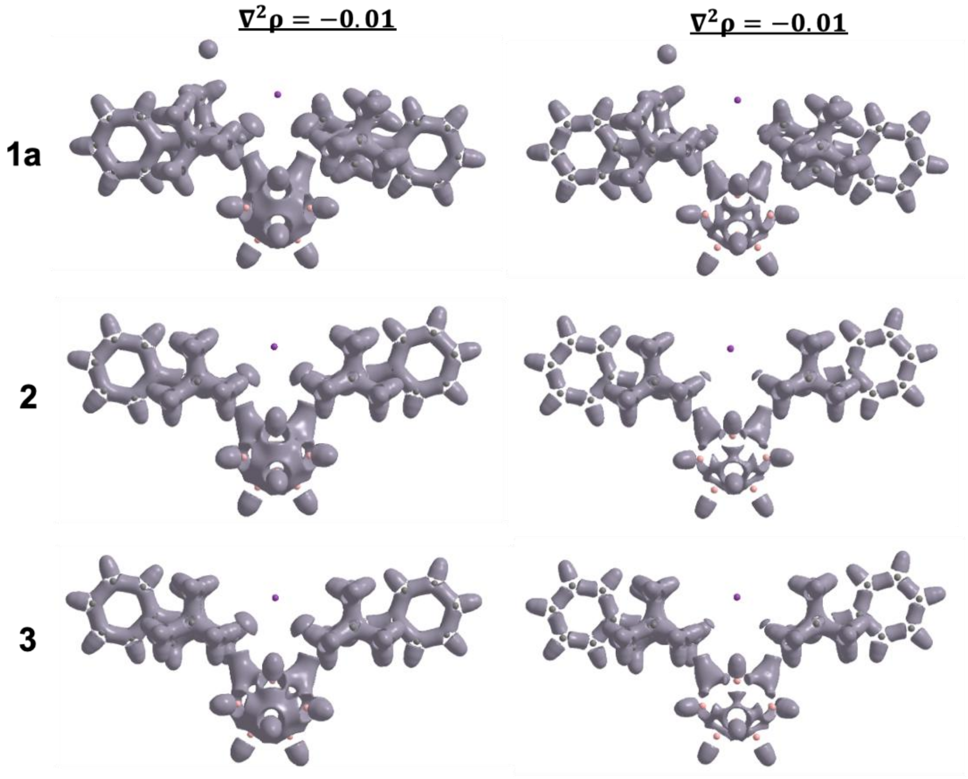

Figure S32: Laplacians of the electron density obtained for the **1a**, **2**, and **3** complexes at the CAM-B3LYP/def2-TZVPD level of theory. Grey, pink, and purple spheres represent the atomic positions of carbon, boron, and bismuth, respectively.

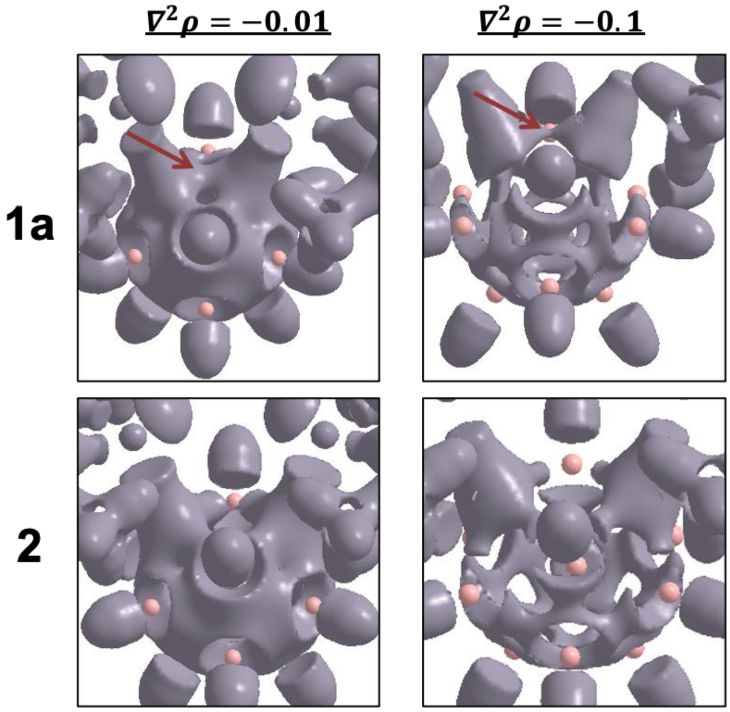

Figure S33: Detail of the $\nabla^2\rho$ at the CAM-B3LYP/def2-TZVPD level of theory between carborane's carbon atoms for compounds **1a** and **2**. Arrows point to the area with negative values of the Laplacian between carborane's carbons in compound 1a, which accounts for the concentration of electron density of the C-C bond. This electron density concentration cannot be seen in compound **2**, in line with the break of this bond that happens upon reduction.

Fragment or Atom	QTAIM Charges	Natural Atomic Charges
**Bi**	-0.31	-0.39
**N-heterocyclic silylenyl (NHSi)**	1.29	1.28
Si	2.06	1.70
NHSi minus Si	-0.77	-0.43
**Carborane**	-2.28	-2.17
$C_{carborane}$	-2.28	-1.00
$B_{10}H_{10}$	2.28	-0.17

Table S11: Charges obtained for compound **2** using different atomic partition formalisms. Electron density distribution is at the CAM-B3LYP/def2-TZVPD level of theory for all situations.

BCP(Si – $C_{carborane}$)	1a	2	3
ρ	1.0850x10^{-1}	1.2629x10^{-1}	1.3908x10^{-1}
$\nabla^2\rho$	1.4587x10^{-1}	1.9320x10^{-1}	2.5821x10^{-1}

Table S12: Electron density (ρ, in a.u. , e-/a_0^3), and Laplacian of the electron density ($\nabla^2\rho$, e-/a_0^5) values at the CAM-B3LYP/def2-TZVPD level of theory for our compounds **1a**, **2**, and **3**. The values reported correspond to the ones at the BCP position between Si and the C atom of the carborane.

B3LYP functional

QTAIM charges/ LI(C_n) & DIs	1a	2	3
Bi	0.09	-0.30	-0.43
C1	-1.73	-2.25	-2.49
C2	-1.72	-2.25	-2.49
B-H cage	1.95	2.27	1.99
Si_1	1.79	2.04	2.09
Si_2	1.91	2.04	2.07
Silylene ligand	1.04	1.27	1.22
C1	5.56	6.15	6.40
C2	5.56	6.15	6.41
B1-C1	0.42	0.46	0.47
C1-C2	0.96	0.37	0.22
B2-C1	0.52	0.56	0.55
Si1-C1	0.49	0.57	0.63
Bi-Si1	1.01	1.00	0.94
B1'-C2	0.42	0.46	0.47
B3-C2	0.51	0.56	0.55
Si2-C2	0.49	0.57	0.63
Bi-Si2	1.03	1.00	0.96

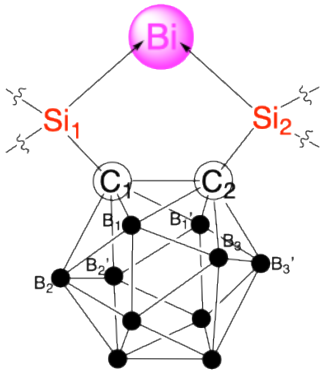

Figure S34: QTAIM charges (in blue) and Localization and Delocalization Indices (LIs & DIs) (in black) of selected atoms of XRD Bismuth complexes **1a**, **2**, and **3** obtained at the B3LYP/def2-TZVPD level of theory. Shaded cells correspond to bonded atom pairs according to the QTAIM analysis. Atom labels follow the classification depicted in the sketch on the right

	**1a**	**2**	**3**
**IP (eV)**	9.05 [a)]	4.54	1.41
**H-L gap (eV)**	3.53 [a)]	1.76	0.92
**β (a.u.)**	---	---	$4.75x10^{+4}$

Table S13: Molecular properties (ionization potential, HOMO-LUMO gap, and first hyperpolarizability, β) computed for the XRD structures of **1a**, **2**, and **3** at the B3LYP/def2-TZVPD level of theory. [a)] Values computed without considering the iodide counterion to permit a direct comparison among the species.

D3. Analysis of Optimized Structures

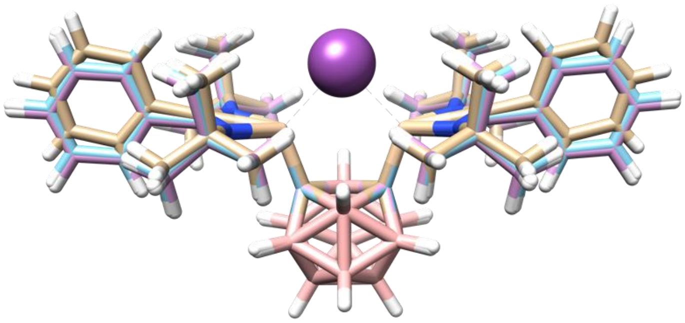

	**XRD**	**OPT (cam-b3lyp)**	**OPT (b3lyp)**
C1-C2	2.2686	2.3329	2.3453
C1-Si_1	1.8515	1.8262	1.8274
C1-B_1	1.7251	1.7068	1.7143
C1-B_2	1.6410	1.6236	1.6281
Si_1-Bi	2.5757	2.5965	2.6131

Figure S35: Comparison between the experimental XRD structure of complex **2** and its computational equivalents optimized at the CAM-B3LYP and B3LYP level of theory. The image on top visually compares the structures: XRD structure holds the C atoms in golden brown, CAM-B3LYP in pink, and B3LYP structure in light blue. The table quantifies the most significative distances in Å.

QTAIM charges/ LI(C_n) & DIs	OPT (CAM-B3LYP)	OPT (B3LYP)
Bi	-0.19	-0.18
C1	-2.45	-2.42
C2	-2.45	-2.42
B-H cage	2.57	2.54
Si_1	2.01	1.99
Si_2	2.01	1.99
Silylene ligand	1.26	1.24
C1	6.36	6.32
C2	6.36	6.32
B1-C1	0.46	0.46
C1-C2	0.36	0.35
B2-C1	0.56	0.56
Si1-C1	0.55	0.56
Bi-Si1	1.01	1.00
B1'-C2	0.46	0.46
B3-C2	0.56	0.56
Si2-C2	0.55	0.56
Bi-Si2	1.01	1.00

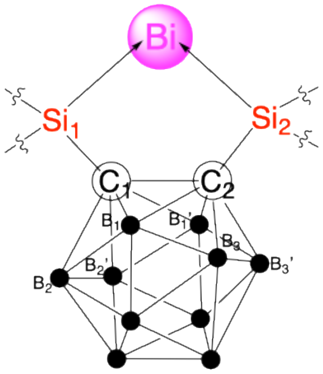

Figure S36: QTAIM charges (in blue) and Localization and Delocalization Indices (LIs & DIs) (in black) of selected atoms of the optimized geometries of the complex **2** at the CAM-B3LYP and B3LYP/def2-TZVPD level of theory. Shaded cells correspond to Bonded atom pairs according to the QTAIM analysIs. Atom labels follow the classification depicted in the sketch on the right

D4. Comments on the non-electride nature

The distinctive and intrinsic characteristic of molecular electrides is the existence of isolated electrons (usually one), and this fact can be unambiguously determined computationally upon the fulfillment of several criteria. The necessary conditions to assert the existence of an isolated electron are the presence of a Non-Nuclear Attractor (NNA), and an ELF (Electron Localization Function) basin or negative values of the Laplacian of the electron density out of nuclear, bond, or valence electron positions.[12] The only presence of basins in valence electron positions, i.e., in the close vicinity of an atom, is insufficient to justify the isolated character that electrides sustain. In this respect, large nonlinear optical properties (NLOPs), in particular, large hyperpolarizabilities, can be used together with the latter conditions to confirm the true electride nature of the given species.

In that sense, we have carried out a complete topological analysis of the electron density of our compounds, identifying the critical points according to QTAIM, and we have also inspected the regions with negative values of the Laplacian of the electron density. In both instances, the features that identify the presence of an isolated electron (NNA and the presence of isolated electron density by the analysis of the Laplacian of the electron density) have not revealed an electride character.

Table S14 below summarizes the characterization of the critical points identified in the topological

analysis, where NNAs do not appear. Poincaré-Hopf relationship is a test to assess the completeness of the topological analysis. It helps identify situations where some critical points are missing and also validates topological analysis results. In turn, Figures S32 and S33 display $\nabla^2\rho$ results for two different negative isovalues. The images do not show a region with negative values at the center of the carborane, which would be compatible with the accumulation of the isolated-electron electron density in that position.

	**1a**	**2**	**3**
**Number of NACPs**	106	105	105
**Number of NNACPs**	**0**	**0**	**0**
**Number of BCPs**	150	139	140
**Number of RCPs**	54	36	38
**Number of CCPs**	9	1	2
**Poincare-Hopf Relationship:**<br>**NACP + NNACP - BCP + RCP - CCP = 1**	✓	✓	✓

Table S14: Summary of the characterization of the critical points identified in the topological analyses of the electron density of the XRD structures of compounds **1a, 2**, and **3**. Electron density generated at the cam-b3lyp/def2-TZVPD level of theory. NACP = Nuclear Attractor Critical Point, NNACP = NonNuclear Attractor Critial Point, BCP = Bond Critical Point, RCP = Ring Critical Point, CCP = Cage Critical Point.

The obtained first hyperpolarizabilities of these compounds (see Table 1 of the main manuscript and Table S14 here) are compatible with the labile, loosely bonded, character of the isolated electron in electrides. However, as the necessary conditions to assert the existence of an isolated electron are not fulfilled, this fact only stresses the compounds hold a moderate-to-high polarizable electron density.

D5. Cartesian Coordinates in Angstrom

Optimized structure of 2

105

NEUTRAL_BisCarborane_OPT-B3LYP.xyz

7	-3.269079000	0.063487000	1.076714000
6	-3.558945000	0.233067000	2.511905000
6	-2.299276000	-0.202147000	3.268690000
1	-2.098013000	-1.266661000	3.113559000
1	-2.435937000	-0.027532000	4.342603000
1	-1.426448000	0.372214000	2.934423000
6	-3.839221000	1.717550000	2.797256000
1	-2.990304000	2.332419000	2.471246000
1	-3.985724000	1.871937000	3.874663000

1	-4.744122000	2.057567000	2.281137000
6	-4.739181000	-0.643416000	2.955968000
1	-5.693860000	-0.290143000	2.554681000
1	-4.807609000	-0.623727000	4.051085000
1	-4.581051000	-1.682234000	2.641101000
5	-1.504934000	-3.165648000	0.891878000
1	-2.474178000	-3.142183000	1.589619000
7	3.269079000	0.063485000	-1.076714000
6	3.558945000	0.233065000	-2.511905000
6	2.299277000	-0.202152000	-3.268690000
1	2.098015000	-1.266666000	-3.113558000
1	2.435937000	-0.027537000	-4.342603000
1	1.426447000	0.372209000	-2.934424000
6	3.839219000	1.717547000	-2.797257000
1	2.990302000	2.332416000	-2.471247000
1	3.985722000	1.871934000	-3.874664000
1	4.744120000	2.057566000	-2.281138000
6	4.739182000	-0.643418000	-2.955967000
1	5.693861000	-0.290142000	-2.554681000
1	4.807610000	-0.623729000	-4.051084000
1	4.581053000	-1.682235000	-2.641100000
5	1.504932000	-3.165650000	-0.891878000
1	2.474176000	-3.142185000	-1.589619000
14	-1.769455000	-0.117574000	-0.000025000
7	-3.269087000	0.063527000	-1.076751000
6	-5.526365000	0.340847000	-0.000021000
6	-6.103912000	1.614417000	-0.000039000
1	-5.466809000	2.497456000	-0.000047000
6	-4.053268000	0.172530000	-0.000015000
6	-7.493755000	1.748456000	-0.000048000
1	-7.940214000	2.742906000	-0.000059000
6	-6.341307000	-0.798658000	-0.000029000
1	-5.881336000	-1.785563000	-0.000023000
6	-8.305985000	0.612355000	-0.000045000
1	-9.391299000	0.718476000	-0.000053000
6	-3.558998000	0.233159000	-2.511926000
6	-2.299340000	-0.201943000	-3.268794000
1	-2.098021000	-1.266457000	-3.113748000
1	-2.436052000	-0.027258000	-4.342689000
1	-1.426524000	0.372437000	-2.934523000
6	-7.728078000	-0.661012000	-0.000038000
1	-8.358988000	-1.549975000	-0.000040000
6	-1.172659000	-1.844725000	-0.000021000
5	0.000023000	-3.936679000	-1.432187000

1	0.000040000	-4.491286000	-2.489214000
6	-3.839372000	1.717641000	-2.797178000
1	-2.990471000	2.332539000	-2.471182000
1	-3.985947000	1.872079000	-3.874568000
1	-4.744262000	2.057578000	-2.280986000
6	-4.739209000	-0.643361000	-2.955980000
1	-5.693885000	-0.290169000	-2.554611000
1	-4.807704000	-0.623600000	-4.051092000
1	-4.581001000	-1.682191000	-2.641195000
5	-1.504904000	-3.165649000	-0.891929000
1	-2.474125000	-3.142185000	-1.589702000
5	-0.883734000	-4.570562000	-0.000015000
1	-1.489295000	-5.598469000	-0.000025000
5	-0.000022000	-2.131450000	1.217186000
1	-0.000041000	-1.419154000	2.166193000
83	0.000001000	1.805291000	0.000000000
14	1.769455000	-0.117575000	0.000025000
7	3.269087000	0.063525000	1.076751000
6	5.526365000	0.340847000	0.000021000
6	6.103911000	1.614417000	0.000039000
1	5.466807000	2.497456000	0.000046000
6	4.053268000	0.172529000	0.000015000
6	7.493753000	1.748458000	0.000048000
1	7.940212000	2.742909000	0.000059000
6	6.341308000	-0.798657000	0.000029000
1	5.881338000	-1.785562000	0.000024000
6	8.305985000	0.612358000	0.000046000
1	9.391299000	0.718480000	0.000054000
6	3.558998000	0.233156000	2.511926000
6	2.299341000	-0.201948000	3.268794000
1	2.098024000	-1.266463000	3.113747000
1	2.436054000	-0.027264000	4.342689000
1	1.426525000	0.372431000	2.934524000
6	7.728079000	-0.661009000	0.000039000
1	8.358990000	-1.549972000	0.000041000
6	1.172658000	-1.844726000	0.000021000
5	-0.000025000	-3.936679000	1.432187000
1	-0.000043000	-4.491286000	2.489214000
6	3.839371000	1.717638000	2.797179000
1	2.990468000	2.332535000	2.471183000
1	3.985945000	1.872076000	3.874569000
1	4.744260000	2.057578000	2.280987000
6	4.739211000	-0.643362000	2.955979000
1	5.693886000	-0.290168000	2.554609000

1	4.807706000	-0.623602000	4.051091000
1	4.581004000	-1.682192000	2.641193000
5	1.504902000	-3.165650000	0.891929000
1	2.474123000	-3.142187000	1.589702000
5	0.883730000	-4.570563000	0.000015000
1	1.489291000	-5.598470000	0.000025000
5	0.000021000	-2.131450000	-1.217186000
1	0.000041000	-1.419154000	-2.166193000

105

NEUTRAL_BisCarborane_OPT-CAM-B3LYP.xyz

7	-3.260922000	0.087067000	1.073472000
6	-3.559573000	0.238900000	2.507340000
6	-2.296293000	-0.163591000	3.265825000
1	-2.068573000	-1.222563000	3.113410000
1	-2.442282000	0.006901000	4.338339000
1	-1.435655000	0.431042000	2.938140000
6	-3.882786000	1.706641000	2.803288000
1	-3.050499000	2.349896000	2.492412000
1	-4.041733000	1.844904000	3.880112000
1	-4.792761000	2.027628000	2.285615000
6	-4.709801000	-0.671435000	2.944627000
1	-5.675895000	-0.342676000	2.551791000
1	-4.774439000	-0.662867000	4.039168000
1	-4.523676000	-1.702681000	2.622661000
5	-1.500218000	-3.127394000	0.886980000
1	-2.469220000	-3.104978000	1.584876000
7	3.260922000	0.087063000	-1.073472000
6	3.559574000	0.238896000	-2.507340000
6	2.296294000	-0.163593000	-3.265825000
1	2.068573000	-1.222564000	-3.113411000
1	2.442283000	0.006899000	-4.338339000
1	1.435657000	0.431040000	-2.938141000
6	3.882789000	1.706637000	-2.803286000
1	3.050502000	2.349894000	-2.492411000
1	4.041736000	1.844901000	-3.880111000
1	4.792763000	2.027623000	-2.285613000
6	4.709802000	-0.671439000	-2.944626000
1	5.675896000	-0.342681000	-2.551789000
1	4.774440000	-0.662871000	-4.039168000
1	4.523675000	-1.702685000	-2.622661000
5	1.500213000	-3.127396000	-0.886981000
1	2.469215000	-3.104982000	-1.584877000
14	-1.766580000	-0.084189000	0.000067000
7	-3.260890000	0.086945000	-1.073384000

6	-5.519185000	0.310232000	0.000053000
6	-6.125209000	1.564174000	0.000105000
1	-5.511515000	2.463544000	0.000114000
6	-4.042447000	0.177178000	0.000033000
6	-7.513247000	1.662818000	0.000151000
1	-7.984607000	2.645100000	0.000184000
6	-6.301275000	-0.845546000	0.000098000
1	-5.817589000	-1.821037000	0.000085000
6	-8.293304000	0.510806000	0.000163000
1	-9.380446000	0.589505000	0.000202000
6	-3.559416000	0.238519000	-2.507307000
6	-2.296062000	-0.164261000	-3.265517000
1	-2.068438000	-1.223213000	-3.112795000
1	-2.441893000	0.005966000	-4.338094000
1	-1.435423000	0.430389000	-2.937864000
6	-7.686031000	-0.743273000	0.000143000
1	-8.294175000	-1.647294000	0.000165000
6	-1.166457000	-1.808990000	0.000057000
5	-0.000067000	-3.895506000	-1.426038000
1	-0.000115000	-4.449280000	-2.483532000
6	-3.882441000	1.706226000	-2.803647000
1	-3.050152000	2.349476000	-2.492764000
1	-4.041172000	1.844260000	-3.880533000
1	-4.792481000	2.027417000	-2.286226000
6	-4.709650000	-0.671826000	-2.944561000
1	-5.675785000	-0.342876000	-2.551993000
1	-4.774087000	-0.663543000	-4.039117000
1	-4.523677000	-1.703005000	-2.622289000
5	-1.500295000	-3.127392000	-0.886846000
1	-2.469360000	-3.104973000	-1.584656000
5	-0.880498000	-4.526931000	0.000038000
1	-1.484131000	-5.555834000	0.000064000
5	0.000056000	-2.098527000	1.211953000
1	0.000110000	-1.387500000	2.163293000
83	0.000002000	1.818755000	0.000000000
14	1.766580000	-0.084192000	-0.000067000
7	3.260891000	0.086941000	1.073384000
6	5.519186000	0.310230000	-0.000053000
6	6.125206000	1.564173000	-0.000103000
1	5.511511000	2.463541000	-0.000110000
6	4.042448000	0.177173000	-0.000033000
6	7.513244000	1.662820000	-0.000149000
1	7.984603000	2.645103000	-0.000181000
6	6.301277000	-0.845547000	-0.000101000

1	5.817594000	-1.821039000	-0.000090000
6	8.293304000	0.510809000	-0.000163000
1	9.380446000	0.589510000	-0.000202000
6	3.559416000	0.238517000	2.507307000
6	2.296061000	-0.164260000	3.265517000
1	2.068435000	-1.223211000	3.112796000
1	2.441891000	0.005969000	4.338094000
1	1.435423000	0.430391000	2.937863000
6	7.686034000	-0.743271000	-0.000146000
1	8.294179000	-1.647291000	-0.000169000
6	1.166455000	-1.808992000	-0.000058000
5	0.000060000	-3.895507000	1.426037000
1	0.000107000	-4.449281000	2.483530000
6	3.882443000	1.706223000	2.803645000
1	3.050154000	2.349474000	2.492760000
1	4.041174000	1.844259000	3.880531000
1	4.792484000	2.027413000	2.286224000
6	4.709648000	-0.671829000	2.944564000
1	5.675784000	-0.342881000	2.551998000
1	4.774083000	-0.663545000	4.039120000
1	4.523675000	-1.703008000	2.622293000
5	1.500290000	-3.127395000	0.886845000
1	2.469355000	-3.104978000	1.584655000
5	0.880490000	-4.526933000	-0.000040000
1	1.484122000	-5.555837000	-0.000066000
5	-0.000059000	-2.098526000	-1.211954000
1	-0.000112000	-1.387500000	-2.163294000

XRD structure of 2

105

Exported from Olex2

N	3.2129	6.0255	4.4833
C	3.4871	5.8058	5.9263
C	2.2932	6.3374	6.6958
H	2.1889	7.2951	6.5161
H	2.4350	6.1987	7.6555
H	1.4843	5.8620	6.4130
C	3.6334	4.3115	6.1787
H	2.8323	3.8462	5.8592
H	3.7427	4.1517	7.1394
H	4.4195	3.9748	5.7000
C	4.7226	6.5713	6.3689

H	5.5231	6.1368	6.0071
H	4.7691	6.5786	7.3478
H	4.6726	7.4921	6.0374
B	1.4941	9.3087	4.3058
H	2.4096	9.3609	4.9488
N	-3.2129	6.0255	2.3321
C	-3.4871	5.8058	0.8891
C	-2.2932	6.3374	0.1196
H	-2.1889	7.2951	0.2993
H	-2.4350	6.1987	-0.8401
H	-1.4843	5.8620	0.4024
C	-3.6334	4.3115	0.6367
H	-2.8323	3.8462	0.9562
H	-3.7427	4.1517	-0.3240
H	-4.4195	3.9748	1.1154
C	-4.7226	6.5713	0.4465
H	-5.5231	6.1368	0.8083
H	-4.7691	6.5786	-0.5324
H	-4.6726	7.4921	0.7780
B	-1.4941	9.3087	2.5096
H	-2.4096	9.3609	1.8666
Si	1.7607	6.2409	3.4077
N	3.2129	6.0255	2.3321
C	5.4447	5.4899	3.4077
C	5.8624	4.1791	3.4077
H	5.2237	3.4758	3.4077
C	3.9827	5.8262	3.4077
C	7.2242	3.8856	3.4077
H	7.5135	2.9808	3.4077
C	6.3801	6.5291	3.4077
H	6.1004	7.4370	3.4077
C	8.1467	4.8966	3.4077
H	9.0734	4.6873	3.4077
C	3.4871	5.8058	0.8891
C	2.2932	6.3374	0.1196
H	2.1889	7.2951	0.2993
H	2.4350	6.1987	-0.8401
H	1.4843	5.8620	0.4024
C	7.7523	6.1928	3.4077
H	8.4054	6.8828	3.4077
C	1.1343	7.9832	3.4077
B	0.0000	10.0941	1.9774
H	0.0000	10.6113	0.9840
C	3.6334	4.3115	0.6367

H	2.8323	3.8462	0.9562
H	3.7427	4.1517	-0.3240
H	4.4195	3.9748	1.1154
C	4.7226	6.5713	0.4465
H	5.5231	6.1368	0.8083
H	4.7691	6.5786	-0.5324
H	4.6726	7.4921	0.7780
B	1.4941	9.3087	2.5096
H	2.4096	9.3609	1.8666
B	0.8884	10.7167	3.4077
H	1.4460	11.6880	3.4077
B	0.0000	8.2824	4.6726
H	0.0000	7.4935	5.4675
Bi	0.0000	4.3610	3.4077
Si	-1.7607	6.2409	3.4077
N	-3.2129	6.0255	4.4833
C	-5.4447	5.4899	3.4077
C	-5.8624	4.1791	3.4077
H	-5.2237	3.4758	3.4077
C	-3.9827	5.8262	3.4077
C	-7.2242	3.8856	3.4077
H	-7.5135	2.9808	3.4077
C	-6.3801	6.5291	3.4077
H	-6.1004	7.4370	3.4077
C	-8.1467	4.8966	3.4077
H	-9.0734	4.6873	3.4077
C	-3.4871	5.8058	5.9263
C	-2.2932	6.3374	6.6958
H	-2.1889	7.2951	6.5161
H	-2.4350	6.1987	7.6555
H	-1.4843	5.8620	6.4130
C	-7.7523	6.1928	3.4077
H	-8.4054	6.8828	3.4077
C	-1.1343	7.9832	3.4077
B	0.0000	10.0941	4.8380
H	0.0000	10.6113	5.8314
C	-3.6334	4.3115	6.1787
H	-2.8323	3.8462	5.8592
H	-3.7427	4.1517	7.1394
H	-4.4195	3.9748	5.7000
C	-4.7226	6.5713	6.3689
H	-5.5231	6.1368	6.0071
H	-4.7691	6.5786	7.3478
H	-4.6726	7.4921	6.0374

B	-1.4941	9.3087	4.3058
H	-2.4096	9.3609	4.9488
B	-0.8884	10.7167	3.4077
H	-1.4460	11.6880	3.4077
B	0.0000	8.2824	2.1428
H	0.0000	7.4935	1.3479

XRD structure of 1

106

Exported from Olex2

Bi	6.4933	1.8087	10.1874
I	3.2635	0.6955	8.6956
Si	8.4752	3.2735	11.0029
Si	5.7181	4.1538	9.3950
N	4.8418	4.5416	7.8362
N	9.1764	3.2878	12.6952
N	10.2418	2.8414	10.8754
N	4.0800	4.8508	9.8243
C	10.3605	2.8970	12.2160
C	3.7614	4.9188	8.5222
C	4.8145	5.6715	5.6104
H	3.9061	6.0073	5.7629
H	4.9536	5.5271	4.6519
H	5.4644	6.3286	5.9393
C	11.5979	2.6896	13.0029
C	4.0214	3.3087	5.8423
H	4.0718	2.5072	6.4035
H	4.2500	3.0740	4.9186
H	3.1121	3.6733	5.8721
C	1.4164	4.5416	7.6968
H	1.5368	3.6011	7.7665
C	5.0018	4.3502	6.3571
C	12.3495	3.8105	13.3228
H	12.0597	4.6733	13.0506
C	8.4251	5.1300	10.4560
C	8.6823	3.3340	14.1169
C	6.4208	3.8286	6.1525
H	7.0639	4.5127	6.4371
H	6.5603	3.6246	5.2043
H	6.5502	3.0163	6.6849
C	11.0504	0.7022	9.9738
H	10.1208	0.4242	9.8394
H	11.3529	0.4134	10.8601
H	11.6156	0.2906	9.2870

C	12.0034	1.4134	13.4093
H	11.4817	0.6480	13.1976
C	2.4624	5.4008	7.9889
C	7.0066	5.5759	9.6516
C	13.5327	3.6661	14.0461
H	14.0503	4.4296	14.2729
C	10.6584	2.6571	8.4925
H	11.1803	2.1968	7.8022
H	10.7697	3.6246	8.3945
H	9.7116	2.4242	8.3966
C	0.1959	5.0596	7.3024
H	-0.5253	4.4712	7.1112
C	9.2664	2.2166	14.9660
H	9.1395	1.3574	14.5102
H	8.8138	2.1950	15.8354
H	10.2246	2.3755	15.1004
C	3.1846	4.8755	11.0292
C	13.1855	1.2888	14.1301
H	13.4752	0.4314	14.4178
C	7.1752	3.1264	14.0358
H	6.7764	3.8412	13.4979
H	6.7945	3.1444	14.9387
H	6.9873	2.2599	13.6197
C	11.1500	2.2257	9.8705
C	12.5964	2.7004	10.0473
H	12.9531	2.3574	10.8937
H	12.6200	3.6805	10.0578
H	13.1424	2.3701	9.3038
C	13.9485	2.4098	14.4304
H	14.7646	2.3105	14.9072
C	2.2956	6.7744	7.8463
H	3.0197	7.3647	8.0206
C	8.9939	4.6842	14.7433
H	9.9651	4.8177	14.7664
H	8.6408	4.7094	15.6569
H	8.5781	5.3954	14.2120
C	0.0146	6.4152	7.1848
H	-0.8311	6.7600	6.9243
B	7.3562	6.7600	8.4659
H	6.7966	6.8575	7.4998
B	9.0352	6.4423	11.3620
H	9.5950	6.3250	12.3260
B	6.6113	7.2131	10.0053
H	5.5644	7.6084	10.0620

B	8.4713	5.4080	8.7620
H	8.6444	4.5975	8.0101
B	7.2892	6.1300	11.2549
H	6.6882	5.7943	12.1370
C	1.0619	7.2780	7.4473
H	0.9395	8.2149	7.3549
B	7.9075	7.7853	11.0848
H	7.7155	8.5669	11.8640
B	9.1058	7.0578	8.5646
H	9.7069	7.3539	7.6657
B	9.4390	7.7040	10.2090
H	10.2610	8.4387	10.4022
B	9.7722	6.0073	9.8352
H	10.8161	5.6065	9.7806
B	7.9520	8.1770	9.3500
H	7.7975	9.2203	8.9720
C	4.0373	5.1138	12.2315
H	4.4796	5.9838	12.1517
H	4.7128	4.4080	12.2987
H	3.4762	5.1065	13.0359
C	2.1480	6.0434	10.9609
H	1.6946	6.1300	11.8241
H	1.4874	5.8538	10.2615
H	2.6132	6.8809	10.7509
C	2.4528	3.5831	11.0995
H	3.0759	2.8683	11.3494
H	2.0608	3.3827	10.2237
H	1.7420	3.6462	11.7695

XRD structure of 3

105

Exported from Olex2

Bi	8.8461	9.6630	6.4853
Si	8.9658	12.2795	6.2890
Si	7.6445	9.5325	4.1680
N	8.1196	8.0935	3.1160
N	8.5531	13.1972	7.8268
N	10.5120	13.0075	6.9813
N	6.1920	8.3862	4.0163
C	6.4735	6.2552	2.6611
C	6.8095	5.0236	3.2122
H	7.2532	4.9792	4.0295
C	8.3595	12.9941	4.7693

C	6.9178	7.5375	3.2689
C	11.9693	12.9390	6.7000
C	10.4104	14.2587	9.1725
C	4.8514	8.2164	4.6145
C	7.4418	11.0841	3.3010
C	12.5880	11.8360	7.5399
H	12.4836	12.0461	8.4704
H	13.5223	11.7604	7.3323
H	12.1490	11.0041	7.3493
C	9.8400	13.4920	8.0401
C	4.5129	9.5137	5.3091
H	5.2048	9.7301	5.9394
H	3.6768	9.4214	5.7715
H	4.4430	10.2169	4.6598
C	5.7761	6.2993	1.4514
H	5.5124	7.1187	1.1022
C	7.4138	13.2101	8.7893
C	10.3941	15.6475	9.1309
H	10.0025	16.0832	8.4081
C	7.6652	12.1615	9.8651
H	6.8801	12.0660	10.4087
H	8.4033	12.4372	10.4143
H	7.8719	11.3204	9.4499
C	10.9718	13.6211	10.2709
H	10.9834	12.6921	10.3181
C	9.3606	7.5910	2.4611
C	10.2714	8.8031	2.3176
H	10.4427	9.1793	3.1858
H	9.8454	9.4603	1.7628
H	11.1014	8.5358	1.9175
C	6.4747	3.8688	2.5328
H	6.6873	3.0432	2.9027
C	12.6704	14.2860	6.9756
H	12.1752	14.9956	6.5604
H	13.5600	14.2618	6.6170
H	12.7107	14.4369	7.9230
C	5.4768	5.1475	0.7719
H	5.0377	5.1883	-0.0472
C	12.1012	12.6003	5.2185
H	11.7347	11.7279	5.0562
H	13.0285	12.6100	4.9712
H	11.6244	13.2501	4.6976
C	11.5191	14.3845	11.3033
H	11.8893	13.9606	12.0431

C	4.9050	7.0807	5.6299
H	5.0764	6.2533	5.1770
H	4.0645	7.0253	6.0923
H	5.6065	7.2505	6.2622
B	8.8239	14.0901	3.6426
H	9.6427	14.8033	3.8143
C	6.1773	12.8156	7.9929
H	6.3277	11.9683	7.5682
H	6.0016	13.4828	7.3248
H	5.4238	12.7481	8.5836
B	7.2212	14.1245	4.3881
H	6.8806	14.8643	5.1279
B	7.4128	14.5153	2.6781
C	10.0214	6.5776	3.3897
H	10.8592	6.2981	3.0141
H	9.4462	5.8157	3.4935
H	10.1739	6.9810	4.2465
C	5.8327	3.9171	1.3136
H	5.6389	3.1305	0.8550
C	10.9521	16.3900	10.1539
H	10.9453	17.3175	10.1067
C	7.2072	14.6074	9.3838
H	6.3592	14.6443	9.8311
H	7.2260	15.2608	8.6799
H	7.9083	14.7951	10.0123
B	6.8415	13.1712	1.6495
B	6.0355	13.4721	3.2273
B	8.8436	12.1162	3.4293
H	9.6656	11.4727	3.7747
C	11.5170	15.7642	11.2353
H	11.8962	16.2667	11.9204
B	7.8233	11.7290	1.8364
H	8.1183	11.0986	0.9852
C	9.0944	6.9930	1.1060
H	8.6246	7.6305	0.5624
H	8.5612	6.2011	1.2041
H	9.9278	6.7680	0.6870
C	3.7893	7.9311	3.5595
H	3.8051	8.6226	2.8933
H	2.9240	7.9068	3.9747
H	3.9691	7.0842	3.1443
B	6.8399	12.2393	4.3560
H	6.5255	11.6875	5.2525
B	6.1953	11.8176	2.5422

H	5.3032	11.2673	2.2082
B	8.6108	13.3165	2.0383
H	6.4377	13.4216	0.6794
H	7.2433	15.5719	2.3403
H	9.3332	13.5911	1.3023
H	5.0079	13.8642	3.3028

References

1. Y. Zhou, S. Raoufmoghaddam, T. Szilvási and M. Driess, *Angew. Chem. Int. Ed.*, 2016, **55**, 12868–12872.
2. G. M. Sheldrick, *Acta Crystallogr. Sect. C Struct. Chem.*, 2015, **71**, 3–8.
3. O. V. Dolomanov, L. J. Bourhis, R. J. Gildea, J. A. K. Howard and H. Puschmann, *J. Appl. Crystallogr.*, 2009, **42**, 339–341.
4. S. Stoll, A. Schweiger, *J. Magn. Reson.* 2006, **178**, 42–55.
5. a) A. D. Becke, *J. Chem. Phys*, 1993, **98**, 5648–5652. b) C. Lee, W. Yang, and R.G. Parr, *Phys. Rev. B*, 1988, **37**, 785.
6. T. Yanai, D. Tew, and N. Handy, *Chem. Phys. Lett*. 2004, **393**, 51–57.
7. a) B. P. Pritchard, D. Altarawy, B. Didier, T.D. Gibsom, and T.L. Windus, *J. Chem. Inf. Model*. 2019, **59**, 4814–4820. b) D. Feller, *J. Comput. Chem*. 1996, **17**, 1571–1586. c) K. L. Schuchardt, B. T. Didier, T. Elsethagen, L. Sun, V. Gurumoorthi, J. Chase, J. Li, and T.L. Windus, *J. Chem. Inf. Model.* 2007, **47**, 1045–1052. d) F. Weigend, and R. Ahlrichs, *Phys. Chem. Chem. Phys.* 2005, **7**, 3297. d) D. Rappoport, F. Furche, *J. Chem. Phys.* 2010, **133**, 134105.
8. M. J. Frisch, G. W. Trucks, H. B. Schlegel, G. E. Scuseria, M. A. Robb, J. R. Cheeseman, G. Scalmani, V. Barone, G. A. Petersson, H. Nakatsuji, X. Li, M. Caricato, A. V. Marenich, J. Bloino, B. G. Janesko, R. Gomperts, B. Mennucci, H. P. Hratchian, J. V. Ortiz, A. F. Izmaylov, J. L. Sonnenberg, Williams, F. Ding, F. Lipparini, F. Egidi, J. Goings, B. Peng, A. Petrone, T. Henderson, D. Ranasinghe, V. G. Zakrzewski, J. Gao, N. Rega, G. Zheng, W. Liang, M. Hada, M. Ehara, K. Toyota, R. Fukuda, J. Hasegawa, M. Ishida, T. Nakajima, Y. Honda, O. Kitao, H. Nakai, T. Vreven, K. Throssell, J. A. Montgomery Jr., J. E. Peralta, F. Ogliaro, M. J. Bearpark, J. J. Heyd, E. N. Brothers, K. N. Kudin, V. N. Staroverov, T. A. Keith, R. Kobayashi, J. Normand, K. Raghavachari, A. P. Rendell, J. C. Burant, S. S. Iyengar, J. Tomasi, M. Cossi, J. M. Millam, M. Klene, C. Adamo, R. Cammi, J. W. Ochterski, R. L. Martin, K. Morokuma, O. Farkas, J. B. Foresman, D. J. Fox, *Gaussian 16 Rev C01* 2016.
9. B. Metz, H. Stoll, and M. Dolg, *J. Chem. Phys.* 2000, **113**, 2563–2569.
10. a) S. Grimme, J. Antony, S. Ehrlich, and H. Krieg, *J. Chem. Phys.* 2010, **132**, 154104. b) S. Grimme, S. Ehrlich, and L. Goerigk, *J. Comput. Chem.* 2011, **32**, 1456–1465.
11. T. A Keith, TK Gristmill Software, Overland Park KS, USA, 2019.
12. V. Postils, M. Garcia-Borràs, M. Solà, J.M. Luis, and E. Matito, *Chem. Commun.* 2021, **125**, 4819–4826.
